# The role of mitochondria in the resistance of melanoma to PD-1 inhibitors

**DOI:** 10.1186/s12967-023-04200-9

**Published:** 2023-05-23

**Authors:** Fei Du, Lu-han Yang, Jiao Liu, Jian Wang, Lianpeng Fan, Suwit Duangmano, Hao Liu, Minghua Liu, Jun Wang, Xiaolin Zhong, Zhuo Zhang, Fang Wang

**Affiliations:** 1 School of Pharmacy, Southwest Medical University, Luzhou, 646000 Sichuan People’s Republic of China; 2 Department of Medical Technology, Faculty of Associated Medical Sciences, Chiang Mai University, Chiang Mai, 50200 Thailand; 3 Department of Pharmacy, Affiliated Hospital of Southwest Medical University, Luzhou, 646000 China

**Keywords:** Mitochondria, Melanoma, PD-1 inhibitor, Drug resistance, Combined therapy

## Abstract

Malignant melanoma is one of the most common tumours and has the highest mortality rate of all types of skin cancers worldwide. Traditional and novel therapeutic approaches, including surgery, targeted therapy and immunotherapy, have shown good efficacy in the treatment of melanoma. At present, the mainstay of treatment for melanoma is immunotherapy combined with other treatment strategies. However, immune checkpoint inhibitors, such as PD-1 inhibitors, are not particularly effective in the clinical treatment of patients with melanoma. Changes in mitochondrial function may affect the development of melanoma and the efficacy of PD-1 inhibitors. To elucidate the role of mitochondria in the resistance of melanoma to PD-1 inhibitors, this review comprehensively summarises the role of mitochondria in the occurrence and development of melanoma, targets related to the function of mitochondria in melanoma cells and changes in mitochondrial function in different cells in melanoma resistant to PD-1 inhibitors. This review may help to develop therapeutic strategies for improving the clinical response rate of PD-1 inhibitors and prolonging the survival of patients by activating mitochondrial function in tumour and T cells.

## Introduction

Melanoma, which is usually referred to as malignant melanoma, is a type of skin cancer that develops from melanocytes. It mostly occurs in the skin, mucous membranes and viscera. Although melanoma accounts for only 7% of total cases of skin cancer, almost 90% of patients die, accounting for 65% of all skin cancer-related deaths [1–3]. Some studies have shown that long-term skin exposure to ultraviolet (UV) light greatly increases the incidence of melanoma and is one of the main factors inducing melanoma [2]. According to statistics, differences in sex and age also affect the incidence of melanoma. The incidence of cutaneous melanoma is higher among men than among women worldwide. For example, in 2020, there were about 325,000 new cases of melanoma (174,000 males and 151,000 females) and 57,000 deaths (32,000 males and 25,000 females) worldwide [4, 5]. Moreover, approximately 40% of patients with malignant melanoma are aged > 65 years, who not only present with more aggressive clinicopathological features but are often diagnosed with advanced cancer [6].

Melanoma is generally classified according to the tumour, node, and metastasis (TNM) staging system: stages I–II, patients with local disease; stage III, lymph node-positive disease; stage IV, advanced/metastatic disease [7]. The key to evaluating the severity of melanoma is the assessment of clinicopathological features such as tumour thickness (Breslow depth), physiological and pathological status of the lymphoid system and ulcers and tumour cell metastasis. The treatment of melanoma varies with the disease stage. In melanoma stages I–II, after the cancer site is determined based on clinical manifestations, surgical resection and adjuvant systemic treatment are used to reduce the risk of recurrence postoperatively [8, 9]. However, patients with lymph node-positive disease or tumour metastasis are not eligible for surgical treatment and may require targeted therapy, immunotherapy or combination therapy for effective treatment [10–13].

Programmed death-1 (PD-1) and its ligand (PD-L1) are co-inhibitory protein receptors expressed on the surface of lymphocytes. Their main physiological functions are maintaining self-tolerance and limiting inflammation in normal tissues [12]. Monoclonal antibodies against PD-1 have demonstrated efficacy in the clinical treatment of melanoma, non-small cell lung cancer and renal cell carcinoma [14] and are one of the most important treatment agents for melanoma [15]. However, anti-PD-1 therapy does not effectively block tumour activity in all patients. Although some patients exhibit a clinical response, a large proportion of these patients may develop acquired resistance after the initial reaction [16].

Mitochondria are key cytoplasmic organelles, which are the main site for aerobic respiration. Some studies have shown that healthy mitochondria can not only inhibit melanoma metastasis but also increase survival. Additionally, activating mitochondrial function can increase the anti-tumour activity of CD8^+^ T cells [17–19].

Based on the relationship among melanoma, PD-1 inhibitors and mitochondria, this review discusses the recent research progress on the role of mitochondria in the treatment of melanoma resistance to PD-1 inhibitors to explore strategies for improving the survival rate of patients through the combination of mitochondrial activation and PD-1 inhibition.

## Resistance mechanism of PD-1 inhibitors in melanoma

Immune checkpoint inhibitor-based therapy is the most effective treatment for metastatic melanoma [20]. To date, three immune checkpoint inhibitors have been approved for the treatment of melanoma: the anti-PD-1 antibodies nivolumab and pembrolizumab and the anti-CTLA-4 antibody ipilimumab [21]. In recent years, PD-1 and PD-L1 inhibitors have been the major focus of research on the immunotherapy of melanoma **(**Table 1**)** [22, 23].

**Table 1:** The current status of immunotherapy for melanoma

Therapeutic method	Reagent	Mechanism	References
CTLA-4inhibitor	Ipilimumab	Anti-CTLA4 antibody	[24, 25]
	Tremelimumab	Anti-CTLA4 antibody	[26]
PD-1 inhibitor	Nivolumab	Anti-PD-1 antibody	[27]
	Pembrolizumab	Anti-PD-1 antibody	[15, 28]
	Cemiplimab	Anti-PD-1 antibody	[29]
PD-L1 inhibitor	Atezolizumab	Anti- PD-L antibody	[30, 31]
	Avelumab	Anti- PD-L antibody	[32]
	Durvalumab	Anti- PD-L antibody	[33]
Others	Interferon-alfa2b and pegylated-interferon-alpha2b	Cytokine activation of T-Cells	[34, 35]
	Talimogene laherparepvec(T-VEC)	Oncolytic virus	[36]
	Relatlimab	Anti- LAG-3 antibody	[37]

PD-1, also known as CD279, is usually expressed on activated T, B and natural killer cells and is highly expressed on tumour-specific T cells [12, 38, 39]. Immune cells expressing PD-1 identify cells as normal by recognising the PD-1 ligand (PD-L1/2) released by the cells, which can suppress the immune system [40]. PD-1 regulates the immune system by inducing apoptosis of mature T cells [41, 42]. In addition, PD-1 can reduce the apoptosis of regulatory T cells, thus limiting the inflammatory response in normal tissues (regulatory T cells are anti-inflammatory cells that inhibit immune responses to self-antigens) [43]. These functions of PD-1 can reduce tissue damage during pathological disturbances such as infection. However, in the tumor microenvironment (TME) of melanoma, after identifying an abnormal antigen in major histocompatibility complex (MHC), T cells release interferon-gamma (IFN-γ) to improve tumour-killing efficiency. IFN-γ released by CD8^+^ T cells can upregulate the expression of PD-L1 on tumour and stromal cells [44]. Simultaneously, T-cell receptor signalling can upregulate the expression of PD-1 on T cells, and the interaction between PD-1 and PD-L1 can lead to misidentification of tumour cells by T cells, thus playing a negative role in the anti-tumour effect [45, 46]. Studies have shown that the regulatory effects of melanoma cells on immune checkpoints can be overcome by using antibodies against PD-1 and PD-L1/2 [14, 47, 48]. In particular, PD-1 inhibitors (nivolumab and pembrolizumab) can effectively block the interaction between PD-1 and its ligands PD-L1 and PD-L2, thus allowing the occurrence of immune responses [28, 49–51]. In addition, studies have shown that the abundance of circulatory PD-1^+^ Tregs (Regulatory T cells) is rapidly decreased, the development of melanoma is significantly inhibited and the risk of metastasis is significantly reduced after anti-PD-1 therapy [52].

**Fig. 1:**
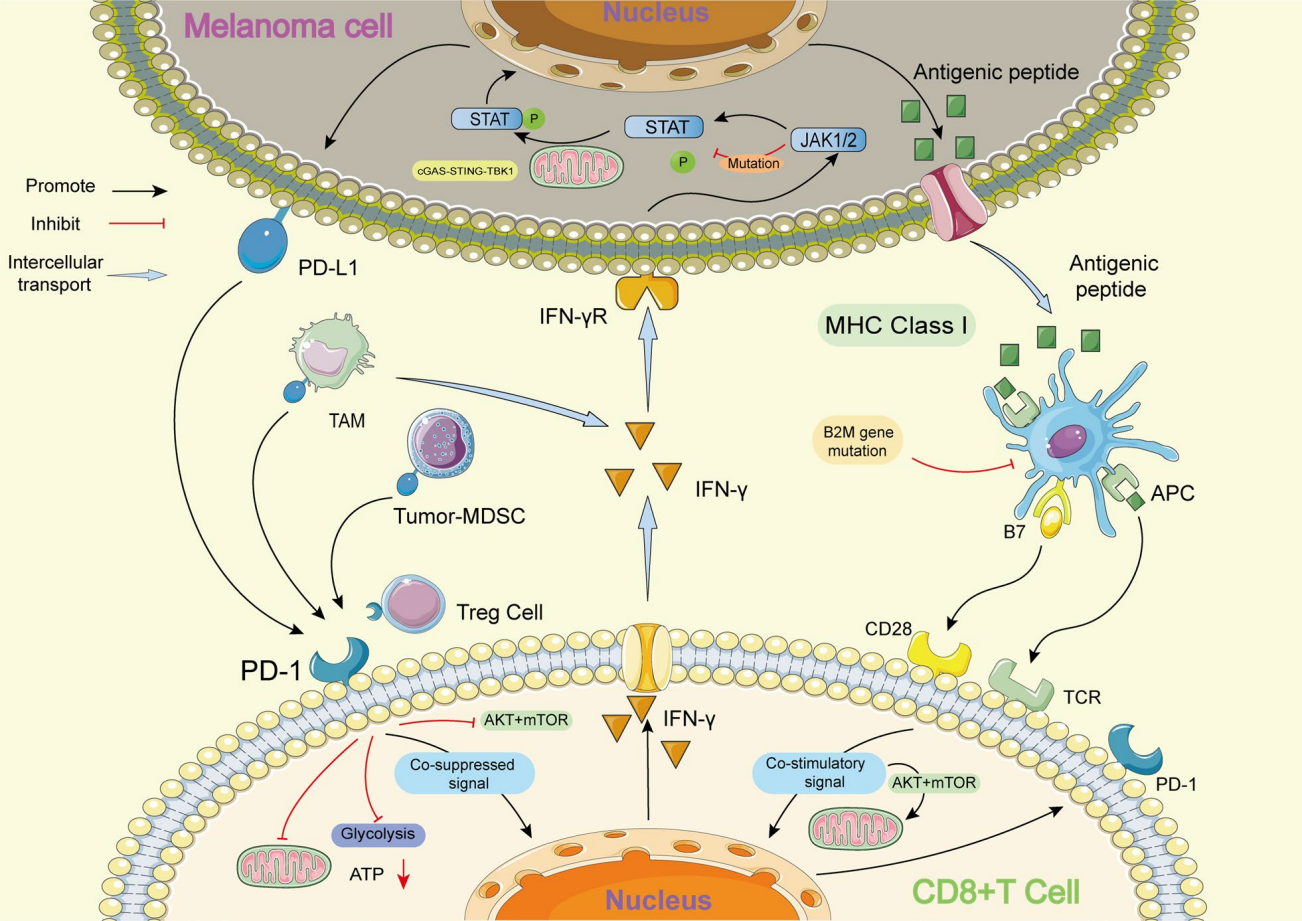
Mechanism of melanoma resistance to PD-1 inhibitors. The main mechanisms of melanoma resistance to PD-1 inhibitors include: (1) Insufficient immunogenicity of tumor; (2) MHC dysfunction caused by B2M mutation; (3) CD8^+^T cells transformed into depleted T cells (Tex) due to continuous stimulation of tumor antigen; (4) IFN- γ signal plays an immunosuppressive role; (5) interference from other cells in tumor microenvironment. MHC: Major histocompatibility complex; B2M: β 2-microglobulin; APC: Antigen-presenting cells; STAT: Signal transducer and activator of transcription; JAK: Janus Kinase; TAM: Tumor-associated macrophages; MDSC: Myeloid-derived suppressor cells

However, most patients cannot benefit from anti-PD-1 therapy, and a large number of patients who respond to anti-PD-1 therapy develop acquired resistance after the initial response in clinical settings [16]. Insufficient tumour immunogenicity, dysfunction of MHC, presence of other inhibitory receptors, IFN-γ signal resistance and an immunosuppressive microenvironment play a key role in the development of resistance to anti-PD-1/PD-L1 therapy (Fig. 1) [16].

### Insufficient immunogenicity of melanoma

The anti-tumour effects of PD-1 blockade depend on the presence of antigen-specific T-cell responses in the tumour microenvironment. They require antigen-presenting cells (APCs) to present potential tumour antigens to activate CD8^+^ T cells and trigger subsequent anti-tumour activity [53]. In a study, patients with melanoma were treated with anti-PD-1 therapy, and the tissue samples of responders and non-responders were subsequently compared. The results showed that patients who were sensitive to anti-PD-1 therapy contained more non-synonymous single nucleotide variants (SNVs) and higher levels of MHC expression products (human leukocyte antigen, HLA) [54]. More new antigens with sufficient immunogenicity are produced in responders, whereas non-responders may be resistant to treatment because of the lack of tumour immunogenicity [55].

### Dysfunction of MHC

Antigen presentation of melanoma cells occurs mainly through the MHCI pathway in TME (tumor cells can activate T cells through MHCI pathway); therefore, tumour cells can evade T cell-mediated killing through the inactivation of MHC-I. β2-microglobulin (B2M) is closely related to the production and stability of the HLAI complex [56]. Some studies have shown that B2M mutation can lead to HLAI dysfunction, which affects antigen presentation and eventually weakens the cytotoxicity of T cells [57]. In a study, after anti-PD-1 therapy, the incidence of B2M gene mutation was three times higher in non-responders than in responders [58]. Therefore, melanoma cells may resist the action of PD-1 inhibitors by interfering with MHC-I function through B2M mutations.

### Emergence of T-cell exhaustion

Owing to the long-term existence of tumour antigens and immunosuppression in TME, T cells may gradually lose their effector function, thus exhibiting a state called ‘exhaustion’. During T-cell exhaustion, effector T cells (Teffs) are also called depleted T cells (Tex) [59]. Studies have shown that under the continuous stimulation of tumour-derived vascular endothelial growth factor (VEGF), CD8^+^ T cells show a state of ‘failure’ and express various inhibitory receptors, including PD-1, T cell immunoglobulin and mucin domain-containing 3 (TIM3), lymphocyte activation gene 3 (LAG3) and cytotoxic T lymphocyte-associated antigen 4 (CTLA4) [60, 61]. In addition, the abundance of PD1 expressed on T cells affects the efficacy of anti-PD-1 therapy, and exhausted CD8^+^ T cells expressing high levels of PD-1 cannot respond to PD-1 inhibitors [62, 63]. This is because terminal Tex is a kind of dysfunctional cells with high expression of inhibitory receptors and little response to specific antigens. Previous studies have suggested that the blockage of PD-1 pathway can reverse terminal Tex in chronic infection into functional T cells [59, 64]. However, subsequent studies have pointed out that the epigenetic state of terminal Tex is difficult to be changed, which means that Immune checkpoint blockade (ICB) can hardly reverse the state of terminal Tex [65].Therefore, T-cell exhaustion is an important phenomenon underlying the resistance to PD-1 inhibitors.

### Resistance to IFN-γ signal

The IFN-γ pathway in tumour cells plays both beneficial and detrimental roles in immunotherapy. T cells produce IFN-γ by recognising receptors on the surface of tumour cells, which can not only increase the expression of MHC molecules to enhance tumour antigen presentation and recruit immune cells but also directly inhibit the proliferation of tumour cells and promote apoptosis [66]. The continuous expression of IFNs can affect the immune editing of tumour cells, which may lead to immunotherapy resistance [67, 68]. In addition, silencing or mutation of genes involved in the IFN-γ pathway, such as JaK1/JaK2 and STATS, can suppress the anti-tumour effects of IFN-γ [69–71] and PD-L1 expression in tumour cells. In such cases, although patients with melanoma with a high tumour mutational burden (TMB) are eligible for anti-PD-1/PD-L1 therapy, anti-PD-1 therapy is not effective. At present, there are two main ways of immunotherapy for IFN- γ pathway. The first is for tumors without IFN- γ signal, which leads to PD-L1 or PD-1 antibody resistance due to the lack of adaptive expression of PD-L1. Activating the alternative interferon pathway (type I IFN) mainly through TLR agonists, oncolytic viruses, or other pathways may also lead to the activation of signal transducer and activator of transcription 1 (STAT1) and STAT2 signals, thus promoting the transcription of PD-L1 and MHC class I by inducing interferon regulatory factor 1 (IRF1) [72]. The second method is for tumors that have been exposed to IFN- γ for a long time (such as ultraviolet-induced malignant melanoma of the skin), which is generally treated with systemic blocking of IFN- γ [73]. Both methods can effectively enhance the efficacy of immune checkpoint inhibitors and reduce their drug resistance.

### Mitochondrial autophagy

Some recent studies have reported that mitochondrial autophagy can affect the role of PD-1 inhibitors. T cells can induce melanoma cell apoptosis by mediating tumour necrosis factor-alpha (TNF-α) signalling. Nuclear factor kappa B (NF-κB) signalling and autophagy play an important role in melanoma cell protection [74, 75]. In a study, T cells were more lethal to melanoma cells after a single autophagy-related gene (CRISPR) was knocked out; however, increased autophagy activity protected tumour cells from T cell-mediated killing [76, 77]. Therefore, targeting mitochondrial autophagy may enhance immunotherapy sensitivity in melanoma.

### Mitochondrial metabolites affect the expression of PD-1 on T cells

Some mitochondrial metabolites in melanoma cells, such as NAD^+^, glucose and glutamine, can promote tumour immune escape. PD-L1 expression has been associated with the content of NAD^+^ and glucose [78, 79]. Glutamine and glucose are indispensable to the development and activation of Teff cells [80]. However, in vivo studies have shown that high expression of PD-1 on the surface of Tregs may be an important factor leading to primary resistance to PD-1 inhibitors [81]. Compared with effector T cells, Treg cells can use Monocarboxylate transporter-1 (MCT1) to efficiently uptake lactic acid in tumor microenvironment with abnormally elevated glycolysis levels, promote the entry of NFAT1 into the nucleus and induce Treg cells to express PD-1, while the expression of PD-1 on CD8^+^T cell will be inhibited, resulting in the failure of PD-1 immunoblocking therapy. This reveals a new way in which lactic acid metabolism affects tumor immune microenvironment, and provides a new idea for developing new tumor immunotherapy strategies targeting lactic acid metabolism (such as MCT1, LDHA) [82, 83].

### Interference of other factors between TME in melanoma

The microenvironment around melanoma cells contains a large number of cytokines, tumour metabolites and immunosuppressive cells that resist the anti-tumour effects of the immune system and promote the growth, reproduction and invasive ability of tumour cells [84, 85]. Tregs in TME can reduce the content of IL-2 in TME and promote the expression of CTLA4 by producing immunosuppressive components such as TGF-β, IL-10 and extracellular adenosine, thus inhibiting the physiological function of CD8^+^ T cells and favouring immune evasion of melanoma cells [86]. Additionally, tumour-associated myeloid-derived suppressor cells (tumour-MDSCs) and tumour-associated macrophages (TAMs) have also been associated with resistance to PD-1 inhibitors. Some studies have found that the expressions of Tyro3, Ax1, MERTK and their ligands of MDSCs in tumor-bearing (melanoma) mice are significantly up-regulated, which can inhibit the ability of T cells to migrate to tumor draining lymph nodes. Interestingly, when drugs inhibit the expression of Tyro3, Ax1 and MERTK in vivo, melanoma growth slows down, CD8^+^T cell infiltration increases, and anti-PD-1 checkpoint inhibitor immunotherapy is enhanced. This suggests that Tyro3, Ax1 and MERTK control the function of MDSC and are expected to be pharmacological targets for regulating MDSC-mediated immunosuppression in patients with melanoma [87]. As a kind of immunosuppressive cells, TAMs can lead to T cell dysfunction and failure by expressing homologous immunoassay ligands (PD-L1) and secreting cytokines and metabolites (such as TGF- β, PGE2) [88–90]. Therefore, TAMS may also be an important target for reversing anti-PD-1 drug resistance.

## Mitochondria and melanoma

Mitochondria are a type of organelle surrounded by two membranes in eukaryotic cells. They produce energy and are the primary site of aerobic respiration. Their diameter is approximately 0.5–1.0 microns. As semi-autonomous organelles, mitochondria have genetic material and a genetic system, namely, mitochondrial DNA (mtDNA); however, their genome size is limited. In addition to producing > 90% ATP to provide energy for cells through oxidative phosphorylation under aerobic conditions, mitochondria are involved in processes such as cell differentiation, information transmission and apoptosis, and can regulate cell growth and cell cycle [91, 92].

**Fig. 2:**
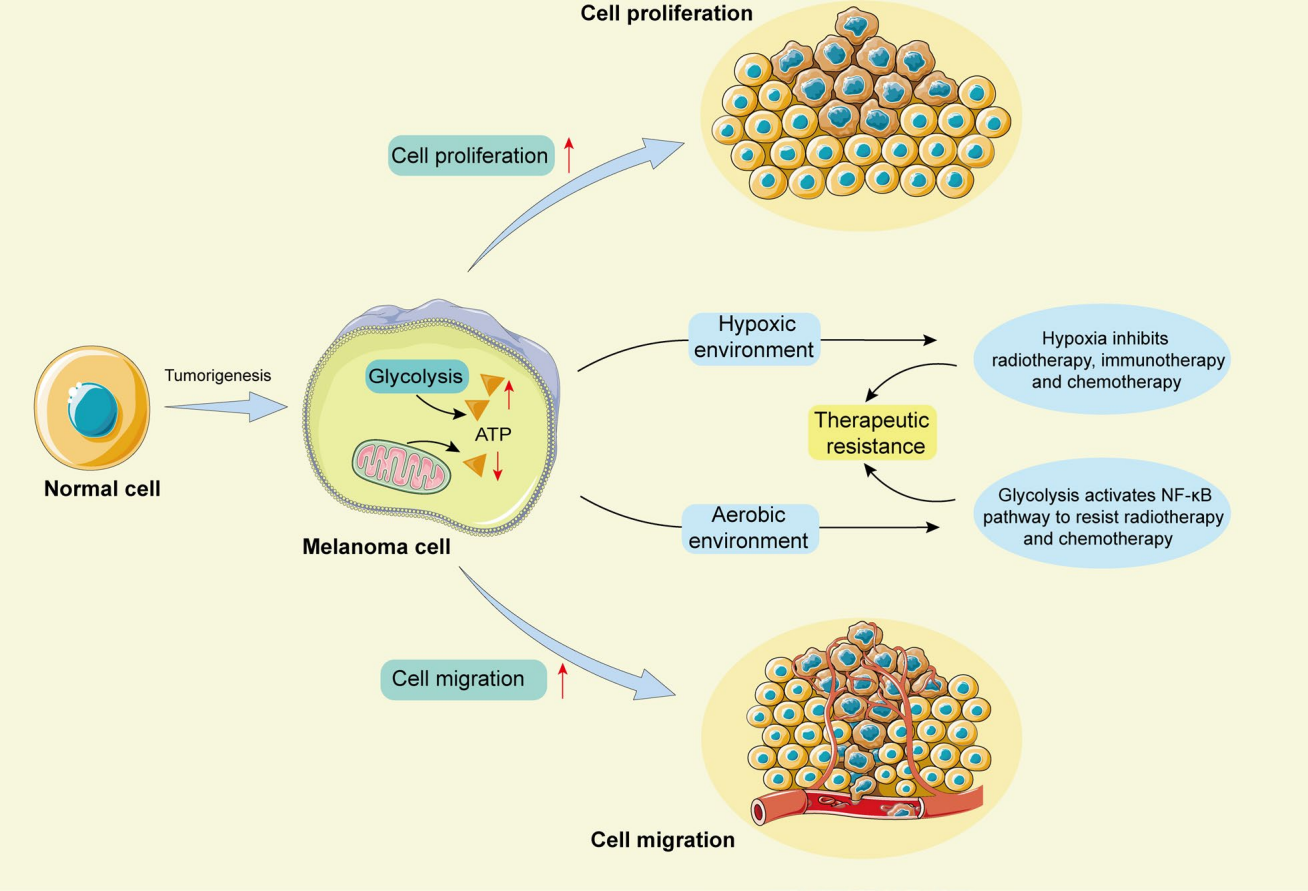
Metabolic changes in melanoma cells affect cell proliferation, migration and therapeutic resistance. When normal cells are transformed into melanoma cells, intracellular mitochondrial metabolism is weakened and glycolysis is enhanced, which will promote tumor cell proliferation, migration and therapeutic resistance

During the occurrence and development of melanoma, the functional changes in mitochondria are related to tumour cell proliferation, metastasis and resistance to targeted therapy [93]. Cells in the tumour centre lack oxygen because they are distant from the blood supply and may be more dependent on glycolysis [94, 95]. Glycolysis may benefit the survival of tumour cells under hypoxic conditions. Moreover, hypoxia can reduce the efficacy of radiotherapy, immunotherapy and chemotherapy [96]. Even under aerobic conditions, the glycolytic pathway in melanoma cells may enhance the resistance to radiation and chemotherapy by activating NF-κB, thus promoting cell survival [97]. Multiple mitochondrial responses and metabolic changes may also promote resistance to targeted therapy [98] (Fig. 2).

**Fig. 3:**
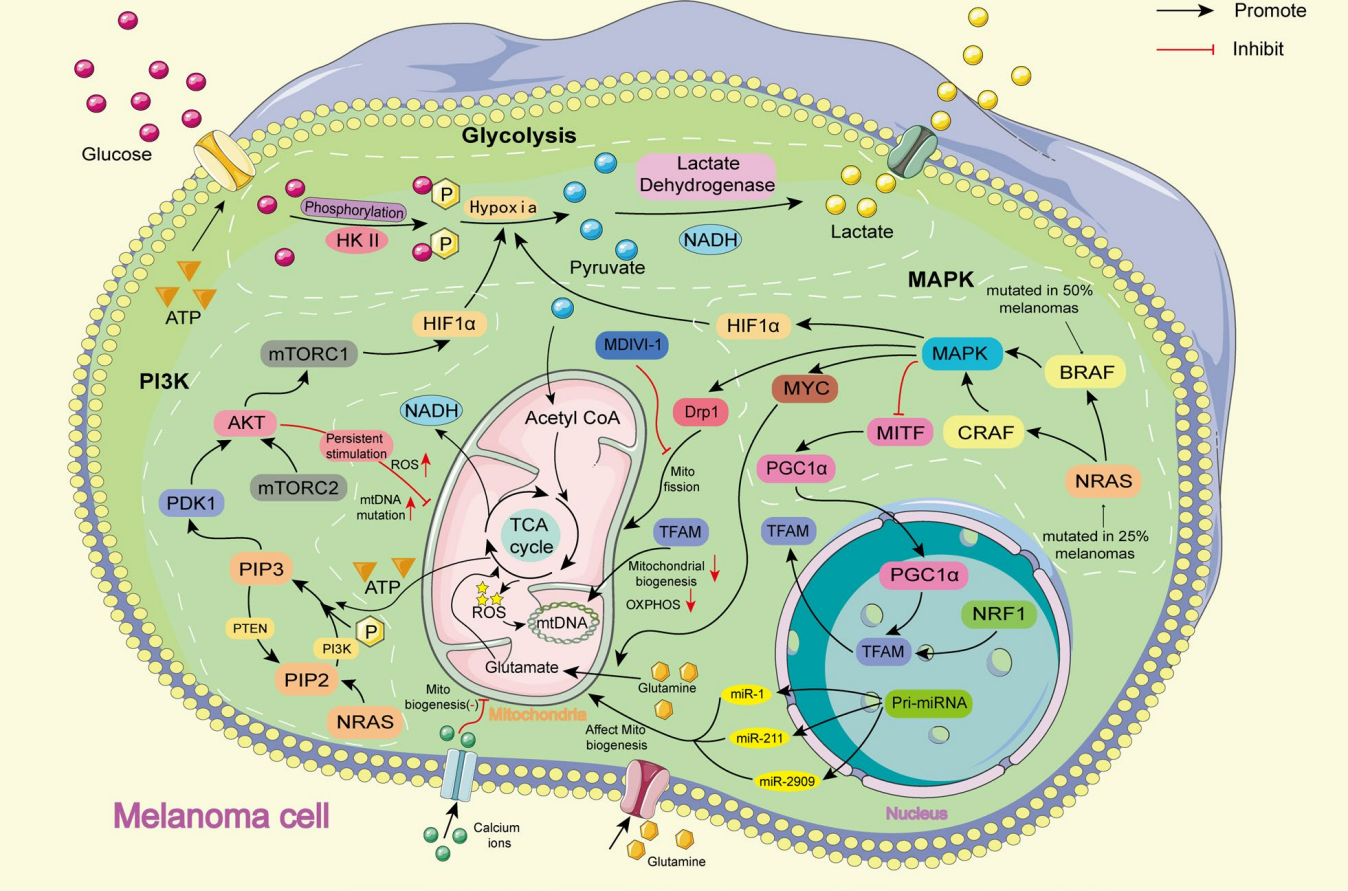
The relationship between the occurrence and development of melanoma cells and mitochondria. Activation and mutation of NRAS or BRAF genes in MAPK and PI3K signaling pathways in melanoma cells can inhibit mitochondrial function and promote glycolysis. The increase of intracellular calcium concentration in melanoma cells can inhibit the function of mitochondria. In addition, some miRNAs may also affect the function of mitochondria. PI3K: Phosphatidylinositol 3-kinase; MAPK: Mitogen-activated protein kinases; NADH: Nicotinamide adenine dinucleotide; HIF1α: Hypoxia inducible factor-1α; mTOR: mammalian Target of rapamycin; PDK1: Phosphoinositide-dependent protein kinase 1; PTEN: Phosphatase and tensin homolog; NRAS: Neuroblastoma RAS; MAPK: Mitogen-activated protein kinase; MITF: Microphthalmia-associated transcription factor; NRF1: Nuclar respiratory factor-1; TFAM: Mitochondrial transcription factor A; MYC: Myelocytomatosis viral oncogene; Drp1: Dynamin-related protein 1; MDIVI-1: Mitochondrial division inhibitor 1

The metabolic changes of tumor cells have always been considered to be closely related to mitochondria [99]. According to the Warburg theory, tumour cells gradually replace mitochondrial oxidative metabolism with glycolysis, thus promoting their rapid proliferation. Melanoma cells often show the characteristics of the classical Warburg effect, with some cells also maintaining high levels of disordered oxidative phosphorylation (OXPHOS). Therefore, inhibition of glycolysis and disordered OXPHOS plays a key role in the treatment of melanoma [100–102]. To date, some drugs with mitochondria-targeting function have been used in experiments or clinics (Table 2). Factors affecting the function of mitochondria in melanoma cells are described below (Fig. 3).

**Table 2:** Studies on the treatment of melanoma by affecting the function of mitochondria

Melanoma type	Reagent	Mechanism	References
MAPK mutation	Talimogene laherparepvec(T-VEC)	Oncolytic virus	[36]
	Prenyl-binding protein phosphodiesterase-δ (PDEδ)	NRAS inhibitor	[103]
	Vemurafenib	BRAF inhibitor	[104]
	Dabrafenib	BRAF inhibitor	[105]
	Encorafenib	BRAF inhibitor	[106]
	Sorafenib	BRAF inhibitor	[107]
	Trametinib	MEK inhibitor	[108]
	Cobimetinib	MEK inhibitor	[109]
	Binimetinib	MEK inhibitor	[110]
mtDNA/DNA mutation	Temozolomide (TMZ)	DNA alkylating agent	[111]
	Fotemustine (FM)	DNA alkylating agent	[112, 113]
	Carboplatin/Cisplatin	DNA alkylating agent	[114, 115]
	Dacarbazine (DTIC)	DNA alkylating agent	[116]
	VAS2870	NADPH oxidase inhibitor, Reduce ROS	[117]
	Diphenylene iodonium (DPI)	NADPH oxidase inhibitor, Reduce ROS	[118, 119]
	Irinotecan	Reduce ROS	[120]
	Apocynin (4-Hydroxy-3-methoxyacetophenone)	PI3K inhibitor, Reduce ROS	[121]
Drp1 expression disorder	Vemurafenib	Inhibition of Drp1 phosphorylation	[122]
	Cryptolepine	Drp1 inhibitor	[123]
	mitochondrial division inhibitor-1 (MDIVI-1)	Drp1 inhibitor	[124, 125]
Calcium signal expression disorder	Diallyl trisulfide	Induce mitochondrial Ca^2+^ overload	[126]
	δ-tocotrienol	Induce mitochondrial Ca^2+^ overload	[127]
	Luteolin	Induce tumor cell death	[128]
	N-acetyl-S-(p-chlorophenylcarbamoyl) cysteine (NACC)	Induce tumor cell death	[129]
	Sanguinarine	Induce tumor cell death	[130]
	Aripiprazole	Depletion of endoplasmic reticulum calcium	[131]
	Digitoxin	Alter mitochondrial membrane potential	[132]
	Lmiquimod	Induce Ca^2+^ depletion	[133, 134]
Abnormal tumor mitochondria OXPHOS	Metformin	OXPHOS inhibitor	[135, 136]
	Atovaquone	OXPHOS inhibitor	[135]
	Arsenic trioxide	OXPHOS inhibitor	[135]
	ONC212	OXPHOS inhibitor	[100]
	Mito-MGN	OXPHOS inhibitor	[101]
	MITO-ONC-RX	OXPHOS inhibitor	[137]
	Chitosan biguanide	Mitochondrial function inhibitor	[138, 139]
	Phenformin	Mitochondrial function inhibitor	[140]
	Sorafenib	Mitochondrial function inhibitor, inducing tumor vessel normalization	[141]

### Mutations in mitochondrial DNA(mtDNA)

There is a set of genetic material in mitochondria independent of nucleus-mtDNA. The length of human mitochondrial DNA is 16569 bp, which has 37 genes and encodes 13 proteins. These proteins are involved in cell energy metabolism [142, 143]. Owing to the lack of protective tissue proteins and related mechanisms of DNA repair, mtDNA has a much higher mutation rate than nuclear DNA under the influence of ROS [144]. Therefore, to maintain their normal function and protect their genome, mitochondria usually respond to genotoxic damage by increasing mtDNA production. [145]. In melanoma cells, the persistent stimulation of mitochondria by AKT protein leads to a decrease in mitochondrial function and an increase in ROS production. Consequently, mtDNA is under oxidative stress for a long time, and ROS can induce oxidative mtDNA (Ox-mtDNA) production [146]. In mitochondria, Ox-mtDNA is either repaired by DNA glycosylase OGG1 or escaped through an open permeability transition pore (mPTP) on the outer membrane of the mitochondria. However, oxidized mtDNA is so large that it needs to be cut into smaller fragments before it can pass through the conversion hole, and this step is done by an enzyme called FEN1. Once cleaved by FEN1, Ox-mtDNA fragments escape through mPTP and enter the cytoplasm, and then they bind to two key factors: nod-like receptor heat protein domain related protein 3 (NLRP3) and cyclic GMP-AMP synthase (cGAS) [147]. The combination of Ox-mtDNA with NLRP3 and cGAS can not only induce inflammation, but also induce the transcription of PD-L1 in melanoma cells, which leads to immune escape. Therefore, we believe that the activation of T cells can be promoted by inhibiting the release of Ox-mtDNA [148, 149]. Some studies have reported that in addition to mtDNA mutations, DNA fixes defects and replication errors also lead to abnormal mitochondrial function and eventually promote the malignant transformation of tumours [150].

Mitochondria are matrilineal organelles that originate from symbiotic bacteria. They coevolve with their hosts, so most mitochondrial proteins are nuclear-encoded. Mitochondrial Lon peptidase 1 (LONP1) is an ATP-dependent protein encoded by nuclear DNA. It is located in the mitochondrial matrix and plays an important role in regulating mitochondrial gene expression and maintaining mitochondrial stability [151]. Some studies have demonstrated that LONP1 expression is significantly increased in melanoma and is related to changes in the metabolic mode of melanoma cells [152]. Overexpression of LONP1 promotes the progression of melanoma, whereas its knockdown inhibits tumour growth and migration. However, if the protein expression of LONP1 is too low, it may lead to mitochondrial dysfunction, thereby promoting the occurrence and development of melanoma [152]. Due to the close communication between mitochondria and nuclei (Cross-talk), in which mtDNA may reverse regulate nuclear gene transcription, the anti-tumor mechanism of mitochondrial therapy may be related to mitochondrial-nuclear interaction. Microarray analysis showed that mitochondria could regulate several carcinogenic pathways, such as cyclin D1, ras, src and p53, and participate in its anti-tumor effect [153, 154].

### Mutations in the BRAF or NRAS gene

During the formation and development of melanoma, mutations in some genes that affect mitochondrial oxidative metabolism may promote the proliferation and survival of melanoma. Activating mutations in the NRAS and BRAF genes play an important role in promoting the progression of melanoma [155].

NRAS gene is located upstream of multiple signalling pathways. Activating mutations in the NRAS gene not only affect the MAPK signalling pathway but also activate PI3K signal transduction [156, 157]. Both MAPK and PI3K pathways eventually promote glycolysis and inhibit mitochondrial oxidative metabolism in melanoma. In addition, clinical studies have shown that melanoma with activating mutations in the NRAS gene is highly invasive and can rapidly develop resistance to existing treatment strategies [155, 158].

Mutation at V600E in the BRAF gene (BRAF^V600E^ mutation) in melanoma cells can promote glycolysis to absorb and integrate substances required for tumour cell proliferation. Additionally, BRAF^V600E^ mutation decreases the expression of PGC-1α by inhibiting the activity of MITF [159]. Some studies have shown that a decrease in the protein expression of PGC-1α can decrease the energy metabolism of mitochondria and increase the content of ROS in the cytoplasm in melanoma. [160]. In addition, BRAF^V600E^–MAPK signalling can regulate mitochondrial function and induce mitochondrial division by activating the expression of dynamin-related protein 1 (Drp1) in melanoma [161].

### Changes in mitochondrial function

The occurrence and development of melanoma is often accompanied by changes in mitochondrial function. In normal somatic cells, a delicate balance exists between mitochondrial division and fusion to maintain proper mitochondrial function [162]. Mitochondrial division is mainly mediated by Drp1, whereas mitochondrial fusion is mediated by dynamic-associated proteins, including Mfn1, Mfn2 and OPA1 [163]. Mitochondrial division in mammalian cells is controlled by Drp1 [161, 164]. During mitochondrial division, Drp1 is transported from the cytoplasm to the mitochondrial outer membrane and binds to fission-1 (Fis1) and mitochondrial fission factor (Mff) located in the mitochondrial outer membrane [165]. During mitochondrial fusion, it is affected by the mitofusin-1/2 gene (Mfn1 and Mfn2) located in the outer membrane of mitochondria and OPA1 in the inner membrane of mitochondria [125]. However, unlike in normal cells, in melanoma cells with activated MAPK mutations, the deletion of Drp1 initiates mitochondrial network reprogramming, induces mitochondrial superfusion and promotes mitochondrial oxidative metabolism [125, 161]. Some studies have shown that mitochondrial division increases in melanoma and mitochondrial fusion may be directly related to chemotherapy resistance [166]. Therefore, changes in mitochondrial dynamics may be a potential target for adjuvant chemotherapy in melanoma.

Mutations in genes involved in the MAPK pathway are most common in melanoma with changes in mitochondrial function, including activating mutations in the BRAF gene, NRAS gene and tyrosine kinase (such as KIT) and inactivating mutation in the NF1 gene [167–170]. These mutations can inhibit the expression of a transcription factor called microphthalmia-associated transcription factor (MITF) in the TME of melanoma. However, MITF can influence the biogenesis and functional regulation of mitochondria by promoting the expression of peroxisome proliferator-activated receptor-γ coactivator-1α (PGC-1α), In addition, the mitochondrial master regulator PGC-1α is associated with the phenotypic transition between proliferation and invasion, which is attributed to different downstream activation signalling pathways [93, 171, 172]. Activation of the PI3K pathway leads to the formation of PIP3. Simultaneously, phosphorylation and activation of AKT by phospholipid-dependent kinase-1 (PDK-1) can directly or indirectly activate mTOR, which in turn induces the expression of hypoxia-inducible factor-1α (HIF1α) [173]. HIF1α inhibits mitochondrial respiration and promotes glycolysis [174]. The mechanisms underlying the occurrence and development of melanoma and related signalling pathways have been identified through large-scale sequencing [175]. Changes in the MAPK and PI3K pathways are more commonly attributed to the occurrence of melanoma and can significantly affect the metabolism of melanoma cells. Additionally, the AMP-activated protein kinase (AMPK) pathway involved in mitochondrial function has been associated with the metabolism of melanoma cells [176, 177]. AMPK is a well-known metabolic receptor that maintains cellular energy homeostasis. It senses the energy content of mitochondria through direct interaction with adenosine triphosphate (ATP), adenosine diphosphate (ADP) and adenosine monophosphate (AMP) [178]. When OXPHOS is inhibited in mitochondria, AMPK can bind to PD-L1 in the endoplasmic reticulum (ER) and phosphorylate PD-L1 at S195, thus reducing the content of PD-L1 in tumour cells [179–181]. In melanoma cells, mutations in a signal pathway can regulate the function of mitochondria, thus affecting the balance of cell metabolism. Therefore, mitochondria play an important role in the occurrence and development of melanoma.

### Other mechanisms

Micro-ribonucleic acids (miRNAs) are promising targets for cancer therapy and may be used as molecular biomarkers of drug resistance in melanoma. Some studies have demonstrated that miR-1 overexpression can induce mitochondrial autophagy in melanoma stem cells (MSCs) by directly targeting the glycerol-3-phosphate dehydrogenase 2 (GPD2) gene and the 3´-UTR of mitochondrial inner membrane tissue system 1 (MINOS1) and interacting with a mitochondrial protein (LRPPRC) [182]. miR-211, which is a tumour suppressor, acts as a metabolic switch and is downregulated in patients with melanoma. Some studies have reported that deletion of miR-211 can lead to downregulation of the TCA cycle and OXPHOS, thereby inhibiting the metabolic function of mitochondria [183]. Additionally, mitochondrial dysfunction associated with the imbalance of miR-2909 expression can lead to the Warburg effect in melanoma [184]. Barbato et al. used integrative genomic analysis and identified the miR-181/TFAM pathway as a key driver of drug resistance in melanoma [185].

Calcium signalling is a major focus of research on anti-tumour therapy. It plays an important role in the pathological status of melanoma. Intracellular calcium concentration, expression of calcium-related signalling proteins, calcium channels on the cell membrane and the Wnt/Ca^2+^ pathway are related to the occurrence and development of melanoma [186, 187]. Evidence documents that increased intracellular calcium stores are associated with highly metastatic melanoma cells [186]. Y-box binding protein 1(YB-1) is an unfavorable prognostic marker secreted from melanoma depending on [Ca^2+^] i and ATP levels, the expression of which increases in primary and metastatic melanoma, compared to benign melanocytic nevi. Interestingly, elevated YB-1 secretion stimulates melanoma cell migration, invasion, and tumorigenicity [188]. It has been reported that Calcium channel dynamics are implicated in melanoma treatment targeting mitochondria stress [189]. Some drugs with anti-melanoma effect (such as diallyl trisulfide) can cause voltage-dependent calcium channel (VDCC)-mediated mitochondrial Ca^2+^ overloa, ROS production and caspase activation, thus inhibiting the biological progression of melanoma [126, 190]. In some conditions, targeting calcium signaling is able to render melanomas more susceptible to conventional therapy, preventing the development of drug resistance and providing novel ideas for combination treatment. For example, tumor necrosis factor-related apoptosis-inducing ligand (TRAIL) is a promising anticancer drug, while some melanomas are resistant to TRAIL therapy. Studies have shown that Ca^2+^ dynamics are a promising approach to overcome TRAIL resistance. Mitochondrial calcium removal can increase the efficacy of TRAIL in the treatment of melanoma through mitochondrial hyperfusion [186]. Therefore, we speculate that targeted calcium signal combined with anti-PD-1 therapy is a potential treatment for melanoma.

## Mitochondria and T cells

Changes in mitochondrial function in melanoma cells can directly affect the development of melanoma, whereas those in immune cells can indirectly affect the progression of melanoma. T lymphocytes are the final effector cells in immune checkpoint inhibitor (ICI)-based immunotherapy, and the number of T cells infiltrating the tumour site is an important indicator of the efficacy of ICI therapy [191, 192]. PD-1 is not only a major regulator of immune homeostasis but also one of the immune checkpoint (IC) molecules [193]. Recent studies have shown that melanoma cells can evade immune surveillance by expressing PD-L1, interacting with PD-1 on T cells and maintaining T cells in a resting state by inhibiting T-cell energy metabolism [194]. In TME, the energy metabolism of mitochondria in T cells plays a key role in regulating anti-tumour immune responses. T cell proliferation requires mitochondrial metabolism, production of ATP for biosynthesis, signal pathways, production of ROS, and activation of NFAT (a key transcription factor produced by IL-2) [195, 196].

Because the metabolism of cells is inseparable from the biological function of mitochondria, the relationship between PD-1 and mitochondria can be recognised from the perspective of cellular bioenergetics. PD-1 is not expressed on immature T cells, which have low metabolic activity and mainly depend on the tricarboxylic acid (TCA) cycle and OXPHOS pathway [197, 198]. During the normal development of T cells, after PD-1 is activated, the metabolism of T cells is temporarily dominated by glycolysis to meet the sudden increase in energy demand [199]. When T cells are required to play an immune-related role, they usually differentiate into Teffs, memory T (TM) cells and Tregs. Teffs (such as CD8^+^ T lymphocytes) can directly kill tumour cells, resulting in upregulation of the TCA cycle and OXPHOS in cells and a decrease in the expression of PD-1 [200, 201]. In an in vitro study, activation of PD-1 decreased the oxygen consumption rate (OCR) and extracellular acidification rate (ECAR), indicating that activation of PD-1 reduced the mitochondrial energy metabolism of T cells [202, 203]. In addition, PD-1 activation can affect mitochondrial function by inhibiting the AKT and mTOR pathways located downstream of TCR [204]. However, owing to the long-term presence of tumour antigens and immunosuppression in TME, T cells may gradually ‘fail’ and lose their function. During T-cell exhaustion, the expression of PD-1 on Teffs is upregulated, and intracellular metabolism shifts to glycolysis [197, 198, 205]. After the TCR–MHC pathway is activated, melanoma-specific CD8^+^ T cells upregulate aerobic glycolysis to support their rapid proliferation and anabolism [206, 207]. Simultaneously, the PI3K–Akt–mTOR signalling pathway is activated, which promotes the secretion of IFN-γ from CD8^+^ T cells, thereby enhancing the anti-tumour function of T cells [208]. However, compared with melanoma cells, T cells have a significantly weak glycolytic ability, which suppresses the anti-tumour function and eventually triggers immune escape [209, 210].

TM cells or Tregs mainly rely on the metabolic pathway of OXPHOS to survive and can inhibit the expression of PD-1 by activating AMPK, thus maintaining the balance of intracellular metabolism [197, 211, 212]. However, contrary to Teff cells and TM cells, Tex cells exhibited metabolic deficiency, including glucose uptake and decreased OXPHOS, some studies have shown that early Tex cells can restore certain proliferation and immune response by activating OXPHOS [213, 214]. In addition, CD8^+^TILs have been shown to lose mitochondrial activity and biogenetic ability due to the decreased expression of transcriptional coactivator PGC-1α, which plays a key role in mitochondrial biogenesis and antioxidation. In B16 mouse tumor model, overexpression of PGC-1α in tumor specific CD8^+^T cells can increase the biogenesis and maintain the effect function of mitochondria, and effectively inhibit tumor activity [214]. Therefore, we speculate that activating the mitochondrial function in effector T cells may inhibit the occurrence of immune escape.

It is well known that calcium channel kinetics is associated with the treatment of melanoma for mitochondrial stress. At present, it has been proved that the increase of [Ca^2+^] i mediated by SOCE can promote the cytotoxicity mediated by cytotoxic T lymphocytes (CTL) in melanoma [215]. However, based on the tumor immune monitoring of T cells, we found that regulatory T cells can inhibit the production of IP3 through TGF- β, reduce the intracellular calcium flow, lead to the death of effector T cells, inhibit the activation of T cells, and induce immunosuppression. Interestingly, when enhancing the high selectivity of calcium signal in CTL or knocking out EGR4 in T cells (EGR4, a member of the zinc finger transcription factor family, was reported as a key regulator of T cell differentiation), IFN- γ production is increased, T cells are activated and melanoma growth is inhibited [216, 217]. Therefore, we speculate that targeting calcium signal combined with anti-PD-1 therapy will be a new hope for melanoma patients.

## Functional changes in mitochondria and resistance of melanoma to PD-1 inhibitors

**Fig. 4:**
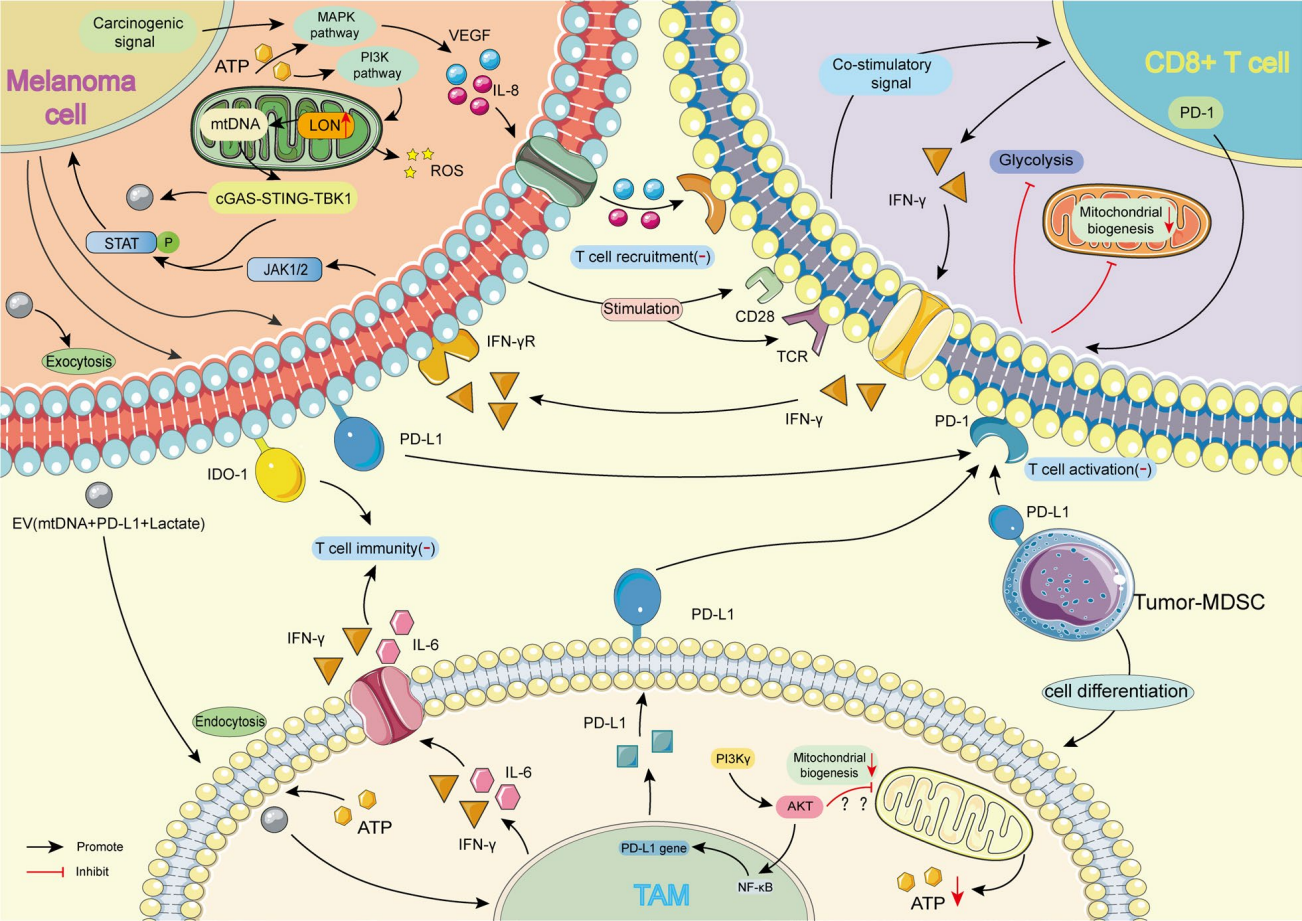
Changes of mitochondrial function in different cells during drug resistance of melanoma TME. Melanoma cells, Tumor-MDSCs, and TAMs can all express PD-L1, which makes CD8^+^ T cells in a resting state. Among them, the expression of PD-L1 in melanoma cells is related to the JAK1/2-STAT pathway involved in mitochondria, while the expression of PD-L1 in TAM may be related to the PI3K γ-NF κ B pathway. When CD8^+^T cells receive dual-signal stimulation from melanoma cells, they can release IFN- γ to participate in immune regulation. After receiving the extracellular carrier (EV) released by melanoma cells, TAMS also releases interferon-γ and IL-6 to inhibit the immune function of T cells. In addition, melanoma cells can inhibit T cell aggregation by producing VEGF and IL-8 through the MAPK pathway, affecting their mitochondria’s function through the PI3K pathway. Tumor-MDSCs: Tumor-associated myeloid-derived suppressor cells; TAM: Tumor-associated macrophages; IFN- γ: Interferon-gamma; VEGF: Vascular endothelial growth factor; IDO-1: Indoleamine 2,3-Dioxygenase-1; EV: Extracellular vesicle

In recent years, anti-PD-1 therapy has emerged as one of the main therapeutic strategies for melanoma. However, most patients do not exhibit a clinical response to PD-1 inhibitors, and those who respond subsequently acquire drug resistance [16, 22, 23]. At present, some researchers have sorted out the effects of internal and external factors of tumor patients on ICB response, drug resistance and toxicity, and put forward the idea of improving the efficacy of ICB by affecting the function of cells in TME [218, 219]. Changes in mitochondrial function in tumour and immune cells in melanoma resistant to PD-1 inhibitors and the effects of other cells in TME on drug resistance are described below (Fig. 4).

### Changes in mitochondrial function in tumour cells

The abnormal expression of some genes and dysfunction of some pathways in tumour cells are internal factors leading to immunotherapy resistance in solid tumour cells (including melanoma). These factors may be active before immunotherapy (primary drug resistance) or may be a result of exposure to immunotherapeutic drugs (acquired drug resistance).

Compared with the metabolism of normal melanocytes, that of melanoma cells is dominated by glycolysis [135]. In the previous sections of this review, we mentioned that changes in mitochondrial energy metabolism in melanoma cells may be one of the causes of drug resistance. Metabolic reprogramming from OXPHOS to glycolysis in tumour cells is caused by the mutation of some genes involved in the MAPK pathway and the continuous expression of HIF1α [220, 221]. Carcinogenic signals can also produce proteins (such as VEGF and IL-8) that inhibit T-cell function through the MAPK pathway [222]. As a result, the recruitment of Teffs to the tumour site and immune responses are suppressed. In addition, when the expression of LON is up-regulated, the metabolic function of mitochondria is inhibited and the production of ROS in mitochondria and the level of oxidative stress are increased. Oxidised mtDNA promotes the signal transduction of IFN-γ through the cGAS–STING–TBK1 pathway in the cytoplasm, which leads to the overexpression of IDO-1 and PD-L1 on the surface of tumour cells to inhibit T-cell activation [149]. Consequently, tumour cells develop immune resistance.

As mentioned earlier, when melanoma cells escape immune surveillance through the PD-L1/PD-1 pathway, PD-L1 expressed on melanoma cells binds to PD-1 on the surface of T cells [40]. Prior to this, the antigen peptides produced by melanoma cells transmit ‘stimulatory’ signals to TCR on the surface of Teffs with the help of APCs. Simultaneously, the B7 family ligands present on APCs bind to CD28 receptors on the surface of Teffs. Under the action of the two types of stimulatory signals, Teffs are activated to kill tumour cells (such as by releasing IFN-γ). Owing to both anti- and pro-cancer functions of IFN-γ, melanoma cells may develop acquired resistance to PD-1 inhibitors [66]. Studies have demonstrated that drug-resistant melanoma cells have lower levels of OXPHOS and lipid metabolism than normal melanoma cells [102]. Therefore, mitochondrial function is further inhibited in melanoma cells resistant to PD-1 inhibitors. These studies suggest that targeted activation of mitochondrial function in melanoma cells combined with anti-PD-1 therapy has become a new treatment strategy for melanoma patients.

### Changes in mitochondrial function in T cells

As mentioned earlier, when T cells are required to exert anti-tumour effects, cellular metabolism dominated by mitochondria not only reduces the expression of PD-1 on the cell surface but also promotes the differentiation of T cells [200, 201]. Differentiated T cells play different roles in the development of drug resistance in melanoma. Therefore, changes in mitochondrial function in T cells may lead to drug resistance in melanoma.

Treg infiltration is found in many human tumours, including melanoma [223]. Tregs can inhibit the response of Teffs by secreting inhibitory factors (such as TGF-β, IL-10 and IL-35) or through cellular contact [224–226]. Therefore, Tregs may play an important role in maintaining auto-tolerance [227]. Several animal studies have reported that primary and adaptive drug resistance to immunotherapy can be reduced by affecting the ratio of Teffs-to-Tregs in TME or by directly removing Tregs [228–230]. An in vivo study demonstrated that Tregs can induce high PD-1 expression in CD8^+^ T cells, leading to primary resistance to PD-1 inhibitors [81]. PD-1 inhibitors usually act on CD8^+^ T cells, which are activated in the draining lymph nodes (DLNs) and transported to the tumour site through the MIG/CXCR3 axis [18, 231]. Activation of CD8^+^ T cells is related to Ca^2+^ release, TCA cycle and ROS production [232–234].

Recognition of PD-L1 on the surface of melanoma cells by PD-1 on the surface of T cells inhibits the metabolism of mitochondria in CD8^+^ T cells. Consequently, CD8^+^ T cells are in a resting state, allowing melanoma cells to escape immune surveillance [194]. However, the co-inhibition of PD-1 transmission in T cells is blocked in melanoma sensitive to PD-1 inhibitors. The costimulatory signal transmitted by TCR activates intracellular mitochondrial function, thus exerting anti-tumour effects [20, 235]. In melanoma resistant to PD-1 inhibitors, CD8^+^ T cells irreversibly differentiate into Tex owing to energy depletion, and other mechanisms of drug resistance may directly or indirectly inhibit mitochondrial function in T cells [59, 236, 237]. Studies have demonstrated that the combination of mitochondria-activating agents, such as uncouplers of mitochondrial OXPHOS (FCCP), mTOR activators, AMPK activators and PGC-1α activators, and PD-1 inhibitors can significantly increase the clinical response rate of PD-1 inhibitors [18, 238–240]. We speculate that an inextricable relationship exists between the decreased immune function of CD8^+^ T cells caused by drug resistance and the inhibited metabolic function of mitochondria in melanoma. To sum up, targeted activation of mitochondrial function in T cells combined with anti-PD-1 therapy can exert a stronger anti-tumor effect and inhibit the occurrence of drug resistance.

### Other cells in TME are involved in the resistance of melanoma to PD-1 inhibitors

In addition to melanoma and common T cells, other cell types in the tumour immune microenvironment are involved in the development of resistance to PD-1 inhibitors. Tumour-MDSCs and TAMs have been associated with resistance to PD-1 inhibitors [87, 241]. At present, the relationship between the resistance of melanoma to PD-1 inhibitors and the changes in mitochondrial function in tumour-MDSCs and TAMs remains unclear. However, both MDSCs and TAMs can participate in the immunosuppressive TME by expressing PD-L1 or producing some cytokines in melanoma [87, 241]. Some studies have demonstrated that inhibition of phosphoinositide 3-kinase (PI3K-γ) in macrophages differentiated from tumour-MDSCs can enhance the immunotherapeutic effects of PD-1 inhibitors in mouse models of melanoma [242, 243]. As mentioned earlier, a correlation exists between the activation of PI3K-related pathways and the inhibition of mitochondrial function. In addition, upregulation of LON in the mitochondria of melanoma cells induces the release of extracellular vesicles (EV) carrying mtDNA, PD-L1 and lactic acid from tumour cells to TAMs, thus inducing TAMs to produce IFN-γ and IL-6. This phenomenon weakens T-cell immunity in TME [149].

To the best of our knowledge, no study has examined the changes in mitochondrial function in tumour-MDSCs and TAMs in melanoma resistant to PD-1 inhibitors. However, given that tumour-MDSCs and TAMs are involved in the development of resistance to PD-1 inhibitors in melanoma, we speculate that functional changes in mitochondria in these two types of cells may be related to PD-1 inhibitor resistance.

## Conclusion and prospect

At present, PD-1 inhibitors are commonly used to treat melanoma; however, their low response rate remains a problem. To reduce the resistance of melanoma to PD-1 inhibitors and improve the response rate, the combination of PD-1 inhibitors and other therapeutic methods is preferred to the independent use of PD-1 inhibitors [244]. The ability of melanoma cells to resist mitochondria-targeting drugs is reduced because of OXPHOS dysfunction. Therefore, the combination of mitochondrial activators and PD-1 inhibitors may represent a new strategy for the treatment of melanoma in the future [102, 245].

In this review, we summarised the mechanisms underlying the resistance of melanoma to PD-1 inhibitors and discussed therapeutic targets related to mitochondrial function in melanoma cells. In addition, we elucidated the changes in mitochondrial function in different cells in melanoma resistant to PD-1 inhibitors. When drug resistance occurs, mitochondria in both melanoma and immune cells are inhibited, and the development of melanoma is not affected. We hypothesised that in melanoma resistant to PD-1 inhibitors, the use of mitochondria-targeting drugs to activate oxidative metabolism in melanoma and immune cells can not only increase the immune activity of CD8^+^ T cells (for example, the release of IFN-γ) but also inhibit tumour progression because of the altered energy metabolism balance by downregulating glycolysis. In 2017, Chamoto et al. demonstrated that mitochondria-activating agents can cooperate with PD-1 inhibitors on T cells, resulting in elevated anti-tumour effects [18]. Therefore, the combination of PD-1 inhibitors and mitochondrial activators in the treatment of melanoma is a very promising research direction.

In conclusion, the tolerance of melanoma to PD-1 inhibitors can be reduced by activating mitochondrial function. However, experimental studies should be conducted to verify this viewpoint. We will continue to investigate the immunological mechanisms underlying the abovementioned combination therapy in future trials and will further describe the clinical benefits of this approach for the treatment of melanoma.

## Data Availability

Not applicable.

## References

[1] Dzwierzynski WW (2013). Managing malignant melanoma. Plast Reconstr Surg.

[2] Ahmed B, Qadir MI, Ghafoor S (2020). Malignant melanoma: skin cancer-diagnosis, prevention, and treatment. Crit Rev Eukaryot Gene Expr.

[3] Filimon A, Preda IA, Boloca AF, Negroiu G (2021). Interleukin-8 in melanoma pathogenesis, prognosis and therapy—an integrated view into other neoplasms and chemokine networks. Cells.

[4] Bellenghi M, Puglisi R, Pontecorvi G, De Feo A, Carè A, Mattia G (2020). Sex and gender disparities in melanoma. Cancers (Basel).

[5] Arnold M, Singh D, Laversanne M, Vignat J, Vaccarella S, Meheus F (2022). Global burden of cutaneous melanoma in 2020 and projections to 2040. JAMA Dermatol.

[6] Iacono D, Vitale MG, Basile D, Pelizzari G, Cinausero M, Poletto E (2021). Immunotherapy for older patients with melanoma: from darkness to light?. Pigment Cell Melanoma Res.

[7] Gershenwald JE, Scolyer RA, Hess KR, Sondak VK, Long GV, Ross MI (2017). Melanoma staging: evidence-based changes in the American joint committee on cancer eighth edition cancer staging manual. CA Cancer J Clin.

[8] Joyce D, Skitzki JJ (2020). Surgical management of primary cutaneous melanoma. Surg Clin North Am.

[9] Eggermont AMM, Robert C, Ribas A (2018). The new era of adjuvant therapies for melanoma. Nat Rev Clin Oncol.

[10] Hu-Lieskovan S, Mok S, Homet Moreno B, Tsoi J, Robert L, Goedert L (2015). Improved antitumor activity of immunotherapy with BRAF and MEK inhibitors in BRAF(V600E) melanoma. Sci Transl Med..

[11] Hauschild A, Grob J-J, Demidov LV, Jouary T, Gutzmer R, Millward M (2012). Dabrafenib in BRAF-mutated metastatic melanoma: a multicentre, open-label, phase 3 randomised controlled trial. Lancet.

[12] Keir ME, Butte MJ, Freeman GJ, Sharpe AH (2008). PD-1 and its ligands in tolerance and immunity. Annu Rev Immunol.

[13] Chapman PB, Hauschild A, Robert C, Haanen JB, Ascierto P, Larkin J (2011). Improved survival with vemurafenib in melanoma with BRAF V600E mutation. N Engl J Med.

[14] Topalian SL, Hodi FS, Brahmer JR, Gettinger SN, Smith DC, McDermott DF (2012). Safety, activity, and immune correlates of anti-PD-1 antibody in cancer. N Engl J Med.

[15] Hamid O, Robert C, Daud A, Hodi FS, Hwu WJ, Kefford R (2019). Five-year survival outcomes for patients with advanced melanoma treated with pembrolizumab in KEYNOTE-001. Ann Oncol.

[16] Lei Q, Wang D, Sun K, Wang L, Zhang Y (2020). Resistance mechanisms of anti-PD1/PDL1 therapy in solid tumors. Front Cell Dev Biol.

[17] Klein K, He K, Younes AI, Barsoumian HB, Chen D, Ozgen T (2020). Role of mitochondria in cancer immune evasion and potential therapeutic approaches. Front Immunol.

[18] Chamoto K, Chowdhury PS, Kumar A, Sonomura K, Matsuda F, Fagarasan S (2017). Mitochondrial activation chemicals synergize with surface receptor PD-1 blockade for T cell-dependent antitumor activity. Proc Natl Acad Sci USA.

[19] Bengsch B, Johnson AL, Kurachi M, Odorizzi PM, Pauken KE, Attanasio J (2016). Bioenergetic insufficiencies due to metabolic alterations regulated by the inhibitory receptor PD-1 are an early driver of CD8(+) T cell exhaustion. Immunity.

[20] Carlino MS, Larkin J, Long GV (2021). Immune checkpoint inhibitors in melanoma. Lancet.

[21] Topalian SL, Drake CG, Pardoll DM (2015). Immune checkpoint blockade: a common denominator approach to cancer therapy. Cancer Cell.

[22] George DD, Armenio VA, Katz SC (2017). Combinatorial immunotherapy for melanoma. Cancer Gene Ther.

[23] Sharma P, Allison JP (2015). Immune checkpoint targeting in cancer therapy: toward combination strategies with curative potential. Cell.

[24] Eggermont AMM, Chiarion-Sileni V, Grob J-J, Dummer R, Wolchok JD, Schmidt H (2016). Prolonged survival in stage III melanoma with ipilimumab adjuvant therapy. N Engl J Med.

[25] Lebbé C, Weber JS, Maio M, Neyns B, Harmankaya K, Hamid O (2014). Survival follow-up and ipilimumab retreatment of patients with advanced melanoma who received ipilimumab in prior phase II studies. Ann Oncol.

[26] Ribas A, Kefford R, Marshall MA, Punt CJA, Haanen JB, Marmol M (2013). Phase III randomized clinical trial comparing tremelimumab with standard-of-care chemotherapy in patients with advanced melanoma. J Clin Oncol.

[27] Robert C, Long GV, Brady B, Dutriaux C, Maio M, Mortier L (2015). Nivolumab in previously untreated melanoma without BRAF mutation. N Engl J Med.

[28] Robert C, Schachter J, Long GV, Arance A, Grob JJ, Mortier L (2015). Pembrolizumab versus ipilimumab in advanced melanoma. N Engl J Med.

[29] Welsh SJ, Corrie PG (2020). Biomarkers predicting for response and relapse with melanoma systemic therapy. Acta Derm Venereol.

[30] Gutzmer R, Stroyakovskiy D, Gogas H, Robert C, Lewis K, Protsenko S (2020). Atezolizumab, vemurafenib, and cobimetinib as first-line treatment for unresectable advanced BRAF mutation-positive melanoma (IMspire150): primary analysis of the randomised, double-blind, placebo-controlled, phase 3 trial. Lancet.

[31] Sullivan RJ, Hamid O, Gonzalez R, Infante JR, Patel MR, Hodi FS (2019). Atezolizumab plus cobimetinib and vemurafenib in BRAF-mutated melanoma patients. Nat Med.

[32] Keilholz U, Mehnert JM, Bauer S, Bourgeois H, Patel MR, Gravenor D (2019). Avelumab in patients with previously treated metastatic melanoma: phase 1b results from the JAVELIN Solid Tumor trial. J Immunother Cancer.

[33] Wang DY, Salem J-E, Cohen JV, Chandra S, Menzer C, Ye F (2018). Fatal toxic effects associated with immune checkpoint inhibitors: a systematic review and meta-analysis. JAMA Oncol.

[34] Eggermont AM, Suciu S, Santinami M, Testori A, Kruit WH, Marsden J (2008). Adjuvant therapy with pegylated interferon alfa-2b versus observation alone in resected stage III melanoma: final results of EORTC 18991, a randomised phase III trial. Lancet.

[35] Davar D, Wang H, Chauvin J-M, Pagliano O, Fourcade JJ, Ka M (2018). Phase Ib/II study of pembrolizumab and pegylated-interferon Alfa-2b in advanced melanoma. J Clin Oncol.

[36] Woller N, Gürlevik E, Ureche C-I, Schumacher A, Kühnel F (2014). Oncolytic viruses as anticancer vaccines. Front Oncol.

[37] Tawbi HA, Schadendorf D, Lipson EJ, Ascierto PA, Matamala L, Castillo Gutiérrez E (2022). Relatlimab and nivolumab versus nivolumab in untreated advanced melanoma. N Engl J Med.

[38] Taube JM, Anders RA, Young GD, Xu H, Sharma R, McMiller TL (2012). Colocalization of inflammatory response with B7–h1 expression in human melanocytic lesions supports an adaptive resistance mechanism of immune escape. Sci Transl Med..

[39] Uhara H (2019). Recent advances in therapeutic strategies for unresectable or metastatic melanoma and real-world data in Japan. Int J Clin Oncol.

[40] Passarelli A, Mannavola F, Stucci LS, Tucci M, Silvestris F (2017). Immune system and melanoma biology: a balance between immunosurveillance and immune escape. Oncotarget.

[41] Dammeijer F, van Gulijk M, Mulder EE, Lukkes M, Klaase L, van den Bosch T (2020). The PD-1/PD-L1-checkpoint restrains T cell immunity in tumor-draining lymph nodes. Cancer Cell.

[42] Sugiura D, Shimizu K, Maruhashi T, Okazaki I-M, Okazaki T (2021). T-cell-intrinsic and -extrinsic regulation of PD-1 function. Int Immunol.

[43] Schatton T, Schütte U, Frank NY, Zhan Q, Hoerning A, Robles SC (2010). Modulation of T-cell activation by malignant melanoma initiating cells. Cancer Res.

[44] Garcia-Diaz A, Shin DS, Moreno BH, Saco J, Escuin-Ordinas H, Rodriguez GA (2017). Interferon receptor signaling pathways regulating PD-L1 and PD-L2 expression. Cell Rep.

[45] Ribas A, Wolchok JD (2018). Cancer immunotherapy using checkpoint blockade. Science.

[46] Akinleye A, Rasool Z (2019). Immune checkpoint inhibitors of PD-L1 as cancer therapeutics. J Hematol Oncol.

[47] Brahmer JR, Tykodi SS, Chow LQM, Hwu W-J, Topalian SL, Hwu P (2012). Safety and activity of anti-PD-L1 antibody in patients with advanced cancer. N Engl J Med.

[48] Lee N, Zakka LR, Mihm MC, Schatton T (2016). Tumour-infiltrating lymphocytes in melanoma prognosis and cancer immunotherapy. Pathology.

[49] Postow MA, Callahan MK, Wolchok JD (2015). Immune checkpoint blockade in cancer therapy. J Clin Oncol.

[50] Ribas A, Puzanov I, Dummer R, Schadendorf D, Hamid O, Robert C (2015). Pembrolizumab versus investigator-choice chemotherapy for ipilimumab-refractory melanoma (KEYNOTE-002): a randomised, controlled, phase 2 trial. Lancet Oncol.

[51] Koller KM, Wang W, Schell TD, Cozza EM, Kokolus KM, Neves RI (2016). Malignant melanoma-The cradle of anti-neoplastic immunotherapy. Crit Rev Oncol Hematol.

[52] Gambichler T, Schröter U, Höxtermann S, Susok L, Stockfleth E, Becker JC (2020). Decline of programmed death-1-positive circulating T regulatory cells predicts more favourable clinical outcome of patients with melanoma under immune checkpoint blockade. Br J Dermatol.

[53] Mempel TR, Henrickson SE, Von Andrian UH (2004). T-cell priming by dendritic cells in lymph nodes occurs in three distinct phases. Nature.

[54] Hugo W, Zaretsky JM, Sun L, Song C, Moreno BH, Hu-Lieskovan S (2016). Genomic and transcriptomic features of response to anti-PD-1 therapy in metastatic melanoma. Cell.

[55] Schumacher TN, Schreiber RD (2015). Neoantigens in cancer immunotherapy. Science.

[56] Hulpke S, Tampé R (2013). The MHC I loading complex: a multitasking machinery in adaptive immunity. Trends Biochem Sci.

[57] Yeon Yeon S, Jung S-H, Jo YS, Choi EJ, Kim MS, Chung Y-J (2019). Immune checkpoint blockade resistance-related B2M hotspot mutations in microsatellite-unstable colorectal carcinoma. Pathol Res Pract.

[58] Sade-Feldman M, Jiao YJ, Chen JH, Rooney MS, Barzily-Rokni M, Eliane J-P (2017). Resistance to checkpoint blockade therapy through inactivation of antigen presentation. Nat Commun.

[59] Wherry EJ, Kurachi M (2015). Molecular and cellular insights into T cell exhaustion. Nat Rev Immunol.

[60] Voron T, Colussi O, Marcheteau E, Pernot S, Nizard M, Pointet A-L (2015). VEGF-a modulates expression of inhibitory checkpoints on CD8+ T cells in tumors. J Exp Med.

[61] Limagne E, Richard C, Thibaudin M, Fumet J-D, Truntzer C, Lagrange A (2019). Tim-3/galectin-9 pathway and mMDSC control primary and secondary resistances to PD-1 blockade in lung cancer patients. Oncoimmunology.

[62] Blackburn SD, Shin H, Freeman GJ, Wherry EJ (2008). Selective expansion of a subset of exhausted CD8 T cells by alphaPD-L1 blockade. Proc Natl Acad Sci USA.

[63] Wherry EJ (2011). T cell exhaustion. Nat Immunol.

[64] Barber DL, Wherry EJ, Masopust D, Zhu B, Allison JP, Sharpe AH (2006). Restoring function in exhausted CD8 T cells during chronic viral infection. Nature.

[65] Liu B, Hu X, Feng K, Gao R, Xue Z, Zhang S (2022). Temporal single-cell tracing reveals clonal revival and expansion of precursor exhausted T cells during anti-PD-1 therapy in lung cancer. Nat Cancer.

[66] Platanias LC (2005). Mechanisms of type-I- and type-II-interferon-mediated signalling. Nat Rev Immunol.

[67] Benci JL, Xu B, Qiu Y, Wu TJ, Dada H, Twyman-Saint Victor C (2016). Tumor interferon signaling regulates a multigenic resistance program to immune checkpoint blockade. Cell.

[68] Shankaran V, Ikeda H, Bruce AT, White JM, Swanson PE, Old LJ (2001). IFNgamma and lymphocytes prevent primary tumour development and shape tumour immunogenicity. Nature.

[69] Darnell JE, Kerr IM, Stark GR (1994). Jak-STAT pathways and transcriptional activation in response to IFNs and other extracellular signaling proteins. Science.

[70] Dunn GP, Bruce AT, Sheehan KCF, Shankaran V, Uppaluri R, Bui JD (2005). A critical function for type I interferons in cancer immunoediting. Nat Immunol.

[71] Kaplan MH, Wurster AL, Grusby MJ (1998). A signal transducer and activator of transcription (Stat)4-independent pathway for the development of T helper type 1 cells. J Exp Med.

[72] Kalbasi A, Ribas A (2020). Tumour-intrinsic resistance to immune checkpoint blockade. Nat Rev Immunol.

[73] Aqbi HF, Wallace M, Sappal S, Payne KK, Manjili MH (2018). IFN-γ orchestrates tumor elimination, tumor dormancy, tumor escape, and progression. J Leukoc Biol.

[74] Vredevoogd DW, Kuilman T, Ligtenberg MA, Boshuizen J, Stecker KE, de Bruijn B (2019). Augmenting immunotherapy impact by lowering tumor TNF cytotoxicity threshold. Cell.

[75] Pan D, Kobayashi A, Jiang P, Ferrari de Andrade L, Tay RE, Luoma AM (2018). A major chromatin regulator determines resistance of tumor cells to T cell-mediated killing. Science.

[76] Young TM, Reyes C, Pasnikowski E, Castanaro C, Wong C, Decker CE (2020). Autophagy protects tumors from T cell-mediated cytotoxicity via inhibition of TNFα-induced apoptosis. Sci Immunol..

[77] Yu Y-R, Imrichova H, Wang H, Chao T, Xiao Z, Gao M (2020). Disturbed mitochondrial dynamics in CD8 TILs reinforce T cell exhaustion. Nat Immunol.

[78] Lv H, Lv G, Chen C, Zong Q, Jiang G, Ye D (2021). NAD metabolism maintains inducible PD-L1 expression to drive tumor immune evasion. Cell Metab.

[79] Guo D, Tong Y, Jiang X, Meng Y, Jiang H, Du L (2022). Aerobic glycolysis promotes tumor immune evasion by hexokinase2-mediated phosphorylation of IκBα. Cell Metab.

[80] Best SA, Gubser PM, Sethumadhavan S, Kersbergen A, Negrón Abril YL, Goldford J (2022). Glutaminase inhibition impairs CD8 T cell activation in STK11-/Lkb1-deficient lung cancer. Cell Metab.

[81] Ngiow SF, Young A, Jacquelot N, Yamazaki T, Enot D, Zitvogel L (2015). A threshold level of intratumor CD8+ T-cell PD1 expression dictates therapeutic response to anti-PD1. Cancer Res.

[82] Kumagai S, Koyama S, Itahashi K, Tanegashima T, Lin Y-T, Togashi Y (2022). Lactic acid promotes PD-1 expression in regulatory T cells in highly glycolytic tumor microenvironments. Cancer Cell.

[83] Tasdogan A, Faubert B, Ramesh V, Ubellacker JM, Shen B, Solmonson A (2020). Metabolic heterogeneity confers differences in melanoma metastatic potential. Nature.

[84] Li X, Wenes M, Romero P, Huang SC, Fendt SM, Ho PC (2019). Navigating metabolic pathways to enhance antitumour immunity and immunotherapy. Nat Rev Clin Oncol.

[85] Gajewski TF, Schreiber H, Fu Y-X (2013). Innate and adaptive immune cells in the tumor microenvironment. Nat Immunol.

[86] Tanaka A, Sakaguchi S (2017). Regulatory T cells in cancer immunotherapy. Cell Res.

[87] Holtzhausen A, Harris W, Ubil E, Hunter DM, Zhao J, Zhang Y (2019). TAM family receptor kinase inhibition reverses MDSC-mediated suppression and augments anti-PD-1 therapy in melanoma. Cancer Immunol Res.

[88] Zhang X, Zeng Y, Qu Q, Zhu J, Liu Z, Ning W (2017). PD-L1 induced by IFN-γ from tumor-associated macrophages via the JAK/STAT3 and PI3K/AKT signaling pathways promoted progression of lung cancer. Int J Clin Oncol.

[89] Lin C, He H, Liu H, Li R, Chen Y, Qi Y (2019). Tumour-associated macrophages-derived CXCL8 determines immune evasion through autonomous PD-L1 expression in gastric cancer. Gut.

[90] Gómez V, Eykyn TR, Mustapha R, Flores-Borja F, Male V, Barber PR (2020). Breast cancer-associated macrophages promote tumorigenesis by suppressing succinate dehydrogenase in tumor cells. Sci Signal..

[91] Annesley SJ, Fisher PR (2019). Mitochondria in health and disease. Cells.

[92] van der Bliek AM, Sedensky MM, Morgan PG (2017). Cell biology of the mitochondrion. Genetics.

[93] Guo W, Wang H, Li C (2021). Signal pathways of melanoma and targeted therapy. Signal Transduct Target Ther.

[94] Ho J, de Moura MB, Lin Y, Vincent G, Thorne S, Duncan LM (2012). Importance of glycolysis and oxidative phosphorylation in advanced melanoma. Mol Cancer.

[95] Kumar PR, Moore JA, Bowles KM, Rushworth SA, Moncrieff MD (2021). Mitochondrial oxidative phosphorylation in cutaneous melanoma. Br J Cancer.

[96] Graham K, Unger E (2018). Overcoming tumor hypoxia as a barrier to radiotherapy, chemotherapy and immunotherapy in cancer treatment. Int J Nanomedicine.

[97] Arbiser JL, Bonner MY, Gilbert LC (2017). Targeting the duality of cancer. NPJ Precis Oncol..

[98] Avagliano A, Fiume G, Pelagalli A, Sanità G, Ruocco MR, Montagnani S (2020). Metabolic plasticity of melanoma cells and their crosstalk with tumor microenvironment. Front Oncol.

[99] Zong W-X, Rabinowitz JD, White E (2016). Mitochondria and cancer. Mol Cell.

[100] Aminzadeh-Gohari S, Weber DD, Catalano L, Feichtinger RG, Kofler B, Lang R (2020). Targeting mitochondria in melanoma. Biomolecules.

[101] Cheng G, Hardy M, Zielonka J, Weh K, Zielonka M, Boyle KA (2020). Mitochondria-targeted magnolol inhibits OXPHOS, proliferation, and tumor growth via modulation of energetics and autophagy in melanoma cells. Cancer Treat Res Commun.

[102] Harel M, Ortenberg R, Varanasi SK, Mangalhara KC, Mardamshina M, Markovits E (2019). Proteomics of melanoma response to immunotherapy reveals mitochondrial dependence. Cell.

[103] Ryan MB, Corcoran RB (2018). Therapeutic strategies to target RAS-mutant cancers. Nat Rev Clin Oncol.

[104] Savoia P, Fava P, Casoni F, Cremona O (2019). Targeting the ERK signaling pathway in melanoma. Int J Mol Sci.

[105] Hauschild A, Ascierto PA, Schadendorf D, Grob JJ, Ribas A, Kiecker F (2020). Long-term outcomes in patients with BRAF V600-mutant metastatic melanoma receiving dabrafenib monotherapy: analysis from phase 2 and 3 clinical trials. Eur J Cancer.

[106] Dummer R, Ascierto PA, Gogas HJ, Arance A, Mandala M, Liszkay G (2018). Encorafenib plus binimetinib versus vemurafenib or encorafenib in patients with BRAF-mutant melanoma (COLUMBUS): a multicentre, open-label, randomised phase 3 trial. Lancet Oncol.

[107] Kim S, Yazici YD, Calzada G, Wang Z-Y, Younes MN, Jasser SA (2007). Sorafenib inhibits the angiogenesis and growth of orthotopic anaplastic thyroid carcinoma xenografts in nude mice. Mol Cancer Ther.

[108] Hoffner B, Benchich K (2018). Trametinib: a targeted therapy in metastatic melanoma. J Adv Pract Oncol.

[109] Ballantyne AD, Garnock-Jones KP (2013). Dabrafenib: first global approval. Drugs.

[110] Dummer R, Schadendorf D, Ascierto PA, Arance A, Dutriaux C, Di Giacomo AM (2017). Binimetinib versus dacarbazine in patients with advanced NRAS-mutant melanoma (NEMO): a multicentre, open-label, randomised, phase 3 trial. Lancet Oncol.

[111] Middleton MR, Grob JJ, Aaronson N, Fierlbeck G, Tilgen W, Seiter S (2000). Randomized phase III study of temozolomide versus dacarbazine in the treatment of patients with advanced metastatic malignant melanoma. J Clin Oncol.

[112] Quéreux G, Dréno B (2011). Fotemustine for the treatment of melanoma. Expert Opin Pharmacother.

[113] Avril MF, Aamdal S, Grob JJ, Hauschild A, Mohr P, Bonerandi JJ (2004). Fotemustine compared with dacarbazine in patients with disseminated malignant melanoma: a phase III study. J Clin Oncol.

[114] Evans LM, Casper ES, Rosenbluth R (1987). Phase II trial of carboplatin in advanced malignant melanoma. Cancer Treat Rep.

[115] Güven K, Kittler H, Wolff K, Pehamberger H (2001). Cisplatin and carboplatin combination as second-line chemotherapy in dacarbazine-resistant melanoma patients. Melanoma Res.

[116] Jiang G, Li R-H, Sun C, Liu Y-Q, Zheng J-N (2014). Dacarbazine combined targeted therapy versus dacarbazine alone in patients with malignant melanoma: a meta-analysis. PLoS ONE.

[117] Moon J-S, Nakahira K, Chung K-P, DeNicola GM, Koo MJ, Pabón MA (2016). NOX4-dependent fatty acid oxidation promotes NLRP3 inflammasome activation in macrophages. Nat Med.

[118] Ghaffarian-Bahraman A, Arabnezhad M-R, Keshavarzi M, Davani-Davari D, Jamshidzadeh A, Mohammadi-Bardbori A (2022). Influence of cellular redox environment on aryl hydrocarbon receptor ligands induced melanogenesis. Toxicol In Vitro.

[119] Chavez-Perez VA, Strasberg-Rieber M, Rieber M (2011). Metabolic utilization of exogenous pyruvate by mutant p53 (R175H) human melanoma cells promotes survival under glucose depletion. Cancer Biol Ther.

[120] Arifa RDN, Paula TPd, Madeira MFM, Lima RL, Garcia ZM, Ÿvila TV (2016). The reduction of oxidative stress by nanocomposite fullerol decreases mucositis severity and reverts leukopenia induced by irinotecan. Pharmacol Res.

[121] Liu J, Zhu H, Premnauth G, Earnest KG, Hahn P, Gray G (2019). UV cell stress induces oxidative cyclization of a protective reagent for DNA damage reduction in skin explants. Free Radic Biol Med.

[122] Ferraz LS, Costa RTd, Costa CAd, Ribeiro CAJ, Arruda DC, Maria-Engler SS (2020). Targeting mitochondria in melanoma: Interplay between MAPK signaling pathway and mitochondrial dynamics. Biochem Pharmacol.

[123] Pal HC, Prasad R, Katiyar SK (2017). Cryptolepine inhibits melanoma cell growth through coordinated changes in mitochondrial biogenesis, dynamics and metabolic tumor suppressor AMPKα1/2-LKB1. Sci Rep.

[124] Akita M, Suzuki-Karasaki M, Fujiwara K, Nakagawa C, Soma M, Yoshida Y (2014). Mitochondrial division inhibitor-1 induces mitochondrial hyperfusion and sensitizes human cancer cells to TRAIL-induced apoptosis. Int J Oncol.

[125] Dal Yontem F, Kim S-H, Ding Z, Grimm E, Ekmekcioglu S, Akcakaya H (2018). Mitochondrial dynamic alterations regulate melanoma cell progression. J Cell Biochem.

[126] Nakagawa C, Suzuki-Karasaki M, Suzuki-Karasaki M, Ochiai T, Suzuki-Karasaki Y (2020). The mitochondrial Ca2+ overload via voltage-gated Ca2+ entry contributes to an anti-melanoma effect of diallyl trisulfide. Int J Mol Sci.

[127] Raimondi M, Fontana F, Marzagalli M, Audano M, Beretta G, Procacci P (2021). Ca overload- and ROS-associated mitochondrial dysfunction contributes to δ-tocotrienol-mediated paraptosis in melanoma cells. Apoptosis.

[128] Kim JK, Kang KA, Ryu YS, Piao MJ, Han X, Oh MC (2016). Induction of endoplasmic reticulum stress via reactive oxygen species mediated by luteolin in melanoma cells. Anticancer Res.

[129] Chen W, Jiang Z, Zhang X, Feng J, Ling Y (2015). N-acetyl-S-(p-chlorophenylcarbamoyl)cysteine induces mitochondrial-mediated apoptosis and suppresses migration in melanoma cells. Oncol Rep.

[130] Burgeiro A, Bento AC, Gajate C, Oliveira PJ, Mollinedo F (2013). Rapid human melanoma cell death induced by sanguinarine through oxidative stress. Eur J Pharmacol.

[131] Forno F, Maatuf Y, Boukeileh S, Dipta P, Mahameed M, Darawshi O (2020). Aripiprazole cytotoxicity coincides with activation of the unfolded protein response in human hepatic cells. J Pharmacol Exp Ther.

[132] Eskiocak U, Ramesh V, Gill JG, Zhao Z, Yuan SW, Wang M (2016). Synergistic effects of ion transporter and MAP kinase pathway inhibitors in melanoma. Nat Commun.

[133] El-Khattouti A, Selimovic D, Hannig M, Taylor EB, Abd Elmageed ZY, Hassan SY (2016). Imiquimod-induced apoptosis of melanoma cells is mediated by ER stress-dependent Noxa induction and enhanced by NF-κB inhibition. J Cell Mol Med.

[134] Nyberg WA, Espinosa A (2016). Imiquimod induces ER stress and Ca(2+) influx independently of TLR7 and TLR8. Biochem Biophys Res Commun.

[135] Ashton TM, McKenna WG, Kunz-Schughart LA, Higgins GS (2018). Oxidative phosphorylation as an emerging target in cancer therapy. Clin Cancer Res.

[136] Peppicelli S, Toti A, Giannoni E, Bianchini F, Margheri F, Del Rosso M (2016). Metformin is also effective on lactic acidosis-exposed melanoma cells switched to oxidative phosphorylation. Cell Cycle.

[137] Sotgia F, Ozsvari B, Fiorillo M, De Francesco EM, Bonuccelli G, Lisanti MP (2018). A mitochondrial based oncology platform for targeting cancer stem cells (CSCs): MITO-ONC-RX. Cell Cycle.

[138] Zhou Z, Zheng C, Liu Y, Luo W, Deng H, Shen J (2022). Chitosan biguanide induced mitochondrial inhibition to amplify the efficacy of oxygen-sensitive tumor therapies. Carbohydr Polym.

[139] Zhou Z, Liu Y, Jiang X, Zheng C, Luo W, Xiang X (2023). Metformin modified chitosan as a multi-functional adjuvant to enhance cisplatin-based tumor chemotherapy efficacy. Int J Biol Macromol.

[140] Zhou Z, Jiang N, Chen J, Zheng C, Guo Y, Ye R (2021). Selectively down-regulated PD-L1 by albumin-phenformin nanoparticles mediated mitochondrial dysfunction to stimulate tumor-specific immunological response for enhanced mild-temperature photothermal efficacy. J Nanobiotechnology.

[141] Zhou Z, Chen J, Liu Y, Zheng C, Luo W, Chen L (2022). Cascade two-stage tumor re-oxygenation and immune re-sensitization mediated by self-assembled albumin-sorafenib nanoparticles for enhanced photodynamic immunotherapy. Acta Pharm Sin B.

[142] Merry TL, Chan A, Woodhead JST, Reynolds JC, Kumagai H, Kim S-J (2020). Mitochondrial-derived peptides in energy metabolism. Am J Physiol Endocrinol Metab.

[143] Riley JS, Tait SW (2020). Mitochondrial DNA in inflammation and immunity. EMBO Rep.

[144] Larsen NB, Rasmussen M, Rasmussen LJ (2005). Nuclear and mitochondrial DNA repair: similar pathways?. Mitochondrion.

[145] Shen J, Gopalakrishnan V, Lee JE, Fang S, Zhao H (2015). Mitochondrial DNA copy number in peripheral blood and melanoma risk. PLoS ONE.

[146] Govindarajan B, Sligh JE, Vincent BJ, Li M, Canter JA, Nickoloff BJ (2007). Overexpression of Akt converts radial growth melanoma to vertical growth melanoma. J Clin Invest.

[147] Xian H, Watari K, Sanchez-Lopez E, Offenberger J, Onyuru J, Sampath H (2022). Oxidized DNA fragments exit mitochondria via mPTP- and VDAC-dependent channels to activate NLRP3 inflammasome and interferon signaling. Immunity.

[148] Theivanthiran B, Evans KS, DeVito NC, Plebanek M, Sturdivant M, Wachsmuth LP (2020). A tumor-intrinsic PD-L1/NLRP3 inflammasome signaling pathway drives resistance to anti-PD-1 immunotherapy. J Clin Invest.

[149] Cheng AN, Cheng L-C, Kuo C-L, Lo YK, Chou H-Y, Chen C-H (2020). Mitochondrial Lon-induced mtDNA leakage contributes to PD-L1-mediated immunoescape via STING-IFN signaling and extracellular vesicles. J Immunother Cancer.

[150] Kopinski PK, Singh LN, Zhang S, Lott MT, Wallace DC (2021). Mitochondrial DNA variation and cancer. Nat Rev Cancer.

[151] Lee YG, Kim HW, Nam Y, Shin KJ, Lee YJ, Park DH (2021). LONP1 and ClpP cooperatively regulate mitochondrial proteostasis for cancer cell survival. Oncogenesis.

[152] Quirós PM, Español Y, Acín-Pérez R, Rodríguez F, Bárcena C, Watanabe K (2014). ATP-dependent Lon protease controls tumor bioenergetics by reprogramming mitochondrial activity. Cell Rep.

[153] Brinker AE, Vivian CJ, Beadnell TC, Koestler DC, Teoh ST, Lunt SY (2020). Mitochondrial haplotype of the host stromal microenvironment alters metastasis in a non-cell autonomous manner. Cancer Res.

[154] Winter E, Dal Pizzol C, Filippin-Monteiro FB, Brondani P, Silva AMPW, Silva AH (2014). Antitumoral activity of a trichloromethyl pyrimidine analogue: molecular cross-talk between intrinsic and extrinsic apoptosis. Chem Res Toxicol.

[155] Randic T, Kozar I, Margue C, Utikal J, Kreis S (2021). NRAS mutant melanoma: towards better therapies. Cancer Treat Rev.

[156] Chen S, Li F, Xu D, Hou K, Fang W, Li Y (2019). The function of RAS mutation in cancer and advances in its drug research. Curr Pharm Des.

[157] Lim SY, Menzies AM, Rizos H (2017). Mechanisms and strategies to overcome resistance to molecularly targeted therapy for melanoma. Cancer.

[158] Johnson DB, Puzanov I (2015). Treatment of NRAS-mutant melanoma. Curr Treat Options Oncol.

[159] Haq R, Shoag J, Andreu-Perez P, Yokoyama S, Edelman H, Rowe GC (2013). Oncogenic BRAF regulates oxidative metabolism via PGC1α and MITF. Cancer Cell.

[160] Vazquez F, Lim J-H, Chim H, Bhalla K, Girnun G, Pierce K (2013). PGC1α expression defines a subset of human melanoma tumors with increased mitochondrial capacity and resistance to oxidative stress. Cancer Cell.

[161] Serasinghe MN, Wieder SY, Renault TT, Elkholi R, Asciolla JJ, Yao JL (2015). Mitochondrial division is requisite to RAS-induced transformation and targeted by oncogenic MAPK pathway inhibitors. Mol Cell.

[162] Pangou E, Sumara I (2021). The multifaceted regulation of mitochondrial dynamics during mitosis. Front Cell Dev Biol.

[163] Sabbah HN (2020). Targeting the mitochondria in heart failure: a translational perspective. JACC Basic Transl Sci..

[164] Jin J-Y, Wei X-X, Zhi X-L, Wang X-H, Meng D (2021). Drp1-dependent mitochondrial fission in cardiovascular disease. Acta Pharmacol Sin.

[165] Zhang Z, Liu L, Wu S, Xing D (2016). Drp1, Mff, Fis1, and MiD51 are coordinated to mediate mitochondrial fission during UV irradiation-induced apoptosis. FASEB J.

[166] Rodrigues T, Ferraz LS (2020). Therapeutic potential of targeting mitochondrial dynamics in cancer. Biochem Pharmacol.

[167] Davies H, Bignell GR, Cox C, Stephens P, Edkins S, Clegg S (2002). Mutations of the BRAF gene in human cancer. Nature.

[168] Held MA, Langdon CG, Platt JT, Graham-Steed T, Liu Z, Chakraborty A (2013). Genotype-selective combination therapies for melanoma identified by high-throughput drug screening. Cancer Discov.

[169] Feng Y, Lau E, Scortegagna M, Ruller C, De SK, Barile E (2013). Inhibition of melanoma development in the Nras((Q61K))::Ink4a(-/-) mouse model by the small molecule BI-69A11. Pigment Cell Melanoma Res.

[170] Krauthammer M, Kong Y, Ha BH, Evans P, Bacchiocchi A, McCusker JP (2012). Exome sequencing identifies recurrent somatic RAC1 mutations in melanoma. Nat Genet.

[171] Li PA, Hou X, Hao S (2017). Mitochondrial biogenesis in neurodegeneration. J Neurosci Res.

[172] Handschin C, Spiegelman BM (2006). Peroxisome proliferator-activated receptor gamma coactivator 1 coactivators, energy homeostasis, and metabolism. Endocr Rev.

[173] Caino MC, Altieri DC (2016). Molecular pathways: mitochondrial reprogramming in tumor progression and therapy. Clin Cancer Res.

[174] Denko NC (2008). Hypoxia, HIF1 and glucose metabolism in the solid tumour. Nat Rev Cancer.

[175] Sullivan RJ, Flaherty K (2013). MAP kinase signaling and inhibition in melanoma. Oncogene.

[176] Schöckel L, Glasauer A, Basit F, Bitschar K, Truong H, Erdmann G (2015). Targeting mitochondrial complex I using BAY 87–2243 reduces melanoma tumor growth. Cancer Metab.

[177] Oscilowska I, Rolkowski K, Baszanowska W, Huynh TYL, Lewoniewska S, Nizioł M (2022). Proline dehydrogenase/proline oxidase (PRODH/POX) is involved in the mechanism of metformin-induced apoptosis in C32 melanoma cell line. Int J Mol Sci.

[178] Deng M, Yang X, Qin B, Liu T, Zhang H, Guo W (2016). Deubiquitination and activation of AMPK by USP10. Mol Cell.

[179] Cha J-H, Yang W-H, Xia W, Wei Y, Chan L-C, Lim S-O (2018). Metformin promotes antitumor immunity via endoplasmic-reticulum-associated degradation of PD-L1. Mol Cell.

[180] Zhou Z, Liu Y, Song W, Jiang X, Deng Z, Xiong W (2022). Metabolic reprogramming mediated PD-L1 depression and hypoxia reversion to reactivate tumor therapy. J Control Release.

[181] Liu Y, Zhou Z, Hou J, Xiong W, Kim H, Chen J (2022). Tumor selective metabolic reprogramming as a prospective PD-L1 depression strategy to reactivate immunotherapy. Adv Mater.

[182] Zhang S, Liu C, Zhang X (2019). Mitochondrial damage mediated by miR-1 overexpression in cancer stem cells. Mol Ther Nucleic Acids.

[183] Mazar J, Qi F, Lee B, Marchica J, Govindarajan S, Shelley J (2016). MicroRNA 211 functions as a metabolic switch in human melanoma cells. Mol Cell Biol.

[184] Kaushik H, Malik D, Parsad D, Kaul D (2019). Mitochondrial respiration is restricted by miR-2909 within human melanocytes. Pigment Cell Melanoma Res.

[185] Barbato A, Iuliano A, Volpe M, D'Alterio R, Brillante S, Massa F (2022). Integrated genomics identifies miR-181/TFAM pathway as a critical driver of drug resistance in melanoma. Int J Mol Sci.

[186] Zhang H, Chen Z, Zhang A, Gupte AA, Hamilton DJ (2022). The role of calcium signaling in melanoma. Int J Mol Sci.

[187] Gajos-Michniewicz A, Czyz M (2020). WNT signaling in melanoma. Int J Mol Sci.

[188] Kosnopfel C, Sinnberg T, Sauer B, Niessner H, Muenchow A, Fehrenbacher B (2020). Tumour progression stage-dependent secretion of YB-1 stimulates melanoma cell migration and invasion. Cancers (Basel)..

[189] Feldman B, Fedida-Metula S, Nita J, Sekler I, Fishman D (2010). Coupling of mitochondria to store-operated Ca(2+)-signaling sustains constitutive activation of protein kinase B/Akt and augments survival of malignant melanoma cells. Cell Calcium.

[190] Rouaud F, Boucher J-L, Slama-Schwok A, Rocchi S (2016). Mechanism of melanoma cells selective apoptosis induced by a photoactive NADPH analogue. Oncotarget.

[191] van der Woude LL, Gorris MAJ, Halilovic A, Figdor CG, de Vries IJM (2017). Migrating into the tumor: a roadmap for T cells. Trends Cancer.

[192] Bonaventura P, Shekarian T, Alcazer V, Valladeau-Guilemond J, Valsesia-Wittmann S, Amigorena S (2019). cold tumors: a therapeutic challenge for immunotherapy. Front Immunol.

[193] Chamoto K, Hatae R, Honjo T (2020). Current issues and perspectives in PD-1 blockade cancer immunotherapy. Int J Clin Oncol.

[194] Tumeh PC, Harview CL, Yearley JH, Shintaku IP, Taylor EJM, Robert L (2014). PD-1 blockade induces responses by inhibiting adaptive immune resistance. Nature.

[195] Siska PJ, Rathmell JC (2015). T cell metabolic fitness in antitumor immunity. Trends Immunol.

[196] Buck MD, O'Sullivan D, Klein Geltink RI, Curtis JD, Chang C-H, Sanin DE (2016). Mitochondrial dynamics controls T cell fate through metabolic programming. Cell.

[197] Li W, Zhang L (2020). Rewiring mitochondrial metabolism for CD8 T cell memory formation and effective cancer immunotherapy. Front Immunol.

[198] Simon S, Labarriere N (2017). PD-1 expression on tumor-specific T cells: friend or foe for immunotherapy?. Oncoimmunology.

[199] Wang R, Green DR (2012). Metabolic checkpoints in activated T cells. Nat Immunol.

[200] van der Windt GJW, Pearce EL (2012). Metabolic switching and fuel choice during T-cell differentiation and memory development. Immunol Rev.

[201] Ahn E, Youngblood B, Lee J, Lee J, Sarkar S, Ahmed R (2016). Demethylation of the PD-1 promoter is imprinted during the effector phase of CD8 T cell exhaustion. J Virol.

[202] Patsoukis N, Bardhan K, Chatterjee P, Sari D, Liu B, Bell LN (2015). PD-1 alters T-cell metabolic reprogramming by inhibiting glycolysis and promoting lipolysis and fatty acid oxidation. Nat Commun.

[203] Patsoukis N, Li L, Sari D, Petkova V, Boussiotis VA (2013). PD-1 increases PTEN phosphatase activity while decreasing PTEN protein stability by inhibiting casein kinase 2. Mol Cell Biol.

[204] Teijeira A, Garasa S, Etxeberria I, Gato-Cañas M, Melero I, Delgoffe GM (2019). Metabolic consequences of T-cell costimulation in anticancer immunity. Cancer Immunol Res.

[205] Sears JD, Waldron KJ, Wei J, Chang C-H (2021). Targeting metabolism to reverse T-cell exhaustion in chronic viral infections. Immunology.

[206] Menk AV, Scharping NE, Moreci RS, Zeng X, Guy C, Salvatore S (2018). Early TCR signaling induces rapid aerobic glycolysis enabling distinct acute T cell effector functions. Cell Rep.

[207] Wang R, Dillon CP, Shi LZ, Milasta S, Carter R, Finkelstein D (2011). The transcription factor Myc controls metabolic reprogramming upon T lymphocyte activation. Immunity.

[208] Salmond RJ (2018). mTOR regulation of glycolytic metabolism in T cells. Front Cell Dev Biol.

[209] Cham CM, Driessens G, O'Keefe JP, Gajewski TF (2008). Glucose deprivation inhibits multiple key gene expression events and effector functions in CD8+ T cells. Eur J Immunol.

[210] Sun N, Tian Y, Chen Y, Guo W, Li C (2022). Metabolic rewiring directs melanoma immunology. Front Immunol.

[211] Le Bourgeois T, Strauss L, Aksoylar H-I, Daneshmandi S, Seth P, Patsoukis N (2018). Targeting T cell metabolism for improvement of cancer immunotherapy. Front Oncol.

[212] Pokhrel RH, Acharya S, Ahn J-H, Gu Y, Pandit M, Kim J-O (2021). AMPK promotes antitumor immunity by downregulating PD-1 in regulatory T cells via the HMGCR/p38 signaling pathway. Mol Cancer.

[213] Gabriel SS, Tsui C, Chisanga D, Weber F, Llano-León M, Gubser PM (2021). Transforming growth factor-β-regulated mTOR activity preserves cellular metabolism to maintain long-term T cell responses in chronic infection. Immunity.

[214] Franco F, Jaccard A, Romero P, Yu Y-R, Ho P-C (2020). Metabolic and epigenetic regulation of T-cell exhaustion. Nat Metab.

[215] Singh K, Rosenberg P (2013). Anti-tumour activity and store operated calcium entry: new roles in immunology. EMBO Mol Med.

[216] Kim K-D, Bae S, Capece T, Nedelkovska H, de Rubio RG, Smrcka AV (2017). Targeted calcium influx boosts cytotoxic T lymphocyte function in the tumour microenvironment. Nat Commun.

[217] Mookerjee-Basu J, Hooper R, Gross S, Schultz B, Go CK, Samakai E (2020). Suppression of Ca2+ signals by EGR4 controls Th1 differentiation and anti-cancer immunity in vivo. EMBO Rep.

[218] Deshpande RP, Sharma S, Watabe K (2020). The confounders of cancer immunotherapy: roles of lifestyle, metabolic disorders and sociological factors. Cancers (Basel)..

[219] Morad G, Helmink BA, Sharma P, Wargo JA (2021). Hallmarks of response, resistance, and toxicity to immune checkpoint blockade. Cell.

[220] Kluza J, Corazao-Rozas P, Touil Y, Jendoubi M, Maire C, Guerreschi P (2012). Inactivation of the HIF-1α/PDK3 signaling axis drives melanoma toward mitochondrial oxidative metabolism and potentiates the therapeutic activity of pro-oxidants. Cancer Res.

[221] Kishton RJ, Barnes CE, Nichols AG, Cohen S, Gerriets VA, Siska PJ (2016). AMPK is essential to balance glycolysis and mitochondrial metabolism to control T-ALL cell stress and survival. Cell Metab.

[222] Liu C, Peng W, Xu C, Lou Y, Zhang M, Wargo JA (2013). BRAF inhibition increases tumor infiltration by T cells and enhances the antitumor activity of adoptive immunotherapy in mice. Clin Cancer Res.

[223] Chaudhary B, Elkord E (2016). Regulatory T cells in the tumor microenvironment and cancer progression: role and therapeutic targeting. Vaccines (Basel)..

[224] Oida T, Zhang X, Goto M, Hachimura S, Totsuka M, Kaminogawa S (2003). CD4+CD25- T cells that express latency-associated peptide on the surface suppress CD4+CD45RBhigh-induced colitis by a TGF-beta-dependent mechanism. J Immunol.

[225] Sakaguchi S, Yamaguchi T, Nomura T, Ono M (2008). Regulatory T cells and immune tolerance. Cell.

[226] Sundstedt A, O'Neill EJ, Nicolson KS, Wraith DC (2003). Role for IL-10 in suppression mediated by peptide-induced regulatory T cells in vivo. J Immunol.

[227] Rudensky AY (2011). Regulatory T cells and Foxp3. Immunol Rev.

[228] Linehan DC, Goedegebuure PS (2005). CD25+ CD4+ regulatory T-cells in cancer. Immunol Res.

[229] Cai J, Wang D, Zhang G, Guo X (2019). The role of PD-1/PD-L1 axis in Treg development and function: implications for cancer immunotherapy. Onco Targets Ther.

[230] Saleh R, Elkord E (2019). Treg-mediated acquired resistance to immune checkpoint inhibitors. Cancer Lett.

[231] Kumar A, Chamoto K, Chowdhury PS, Honjo T (2020). Tumors attenuating the mitochondrial activity in T cells escape from PD-1 blockade therapy. Elife.

[232] Uzhachenko R, Shanker A, Yarbrough WG, Ivanova AV (2015). Mitochondria, calcium, and tumor suppressor Fus1: at the crossroad of cancer, inflammation, and autoimmunity. Oncotarget.

[233] Turrens JF (2003). Mitochondrial formation of reactive oxygen species. J Physiol.

[234] Weinberg SE, Sena LA, Chandel NS (2015). Mitochondria in the regulation of innate and adaptive immunity. Immunity.

[235] Siddiqui I, Schaeuble K, Chennupati V, Fuertes Marraco SA, Calderon-Copete S, Pais Ferreira D (2019). Intratumoral Tcf1PD-1CD8 T cells with stem-like properties promote tumor control in response to vaccination and checkpoint blockade immunotherapy. Immunity.

[236] Bagchi S, Yuan R, Engleman EG (2021). Immune checkpoint inhibitors for the treatment of cancer: clinical impact and mechanisms of response and resistance. Annu Rev Pathol.

[237] Shergold AL, Millar R, Nibbs RJB (2019). Understanding and overcoming the resistance of cancer to PD-1/PD-L1 blockade. Pharmacol Res.

[238] Inoki K, Kim J, Guan K-L (2012). AMPK and mTOR in cellular energy homeostasis and drug targets. Annu Rev Pharmacol Toxicol.

[239] Berrien-Elliott MM, Yuan J, Swier LE, Jackson SR, Chen CL, Donlin MJ (2015). Checkpoint blockade immunotherapy relies on T-bet but not Eomes to induce effector function in tumor-infiltrating CD8+ T cells. Cancer Immunol Res.

[240] Scharping NE, Menk AV, Moreci RS, Whetstone RD, Dadey RE, Watkins SC (2016). The tumor microenvironment represses T cell Mitochondrial biogenesis to drive intratumoral T cell metabolic insufficiency and dysfunction. Immunity.

[241] Hartley GP, Chow L, Ammons DT, Wheat WH, Dow SW (2018). Programmed cell death ligand 1 (PD-L1) signaling regulates macrophage proliferation and activation. Cancer Immunol Res.

[242] De Henau O, Rausch M, Winkler D, Campesato LF, Liu C, Cymerman DH (2016). Overcoming resistance to checkpoint blockade therapy by targeting PI3Kγ in myeloid cells. Nature.

[243] Kaneda MM, Messer KS, Ralainirina N, Li H, Leem CJ, Gorjestani S (2016). PI3Kγ is a molecular switch that controls immune suppression. Nature.

[244] Hayashi H, Nakagawa K (2020). Combination therapy with PD-1 or PD-L1 inhibitors for cancer. Int J Clin Oncol.

[245] Menk AV, Scharping NE, Rivadeneira DB, Calderon MJ, Watson MJ, Dunstane D (2018). 4–1BB costimulation induces T cell mitochondrial function and biogenesis enabling cancer immunotherapeutic responses. J Exp Med.

